# Supplementary figures and images for: Single-cell transcriptome landscape of ovarian cells during primordial follicle assembly in mice
Source: PLoS Biol. 2020 Dec 22;18(12):e3001025. doi: 10.1371/journal.pbio.3001025 (PMC7787681; doi:10.1371/journal.pbio.3001025)

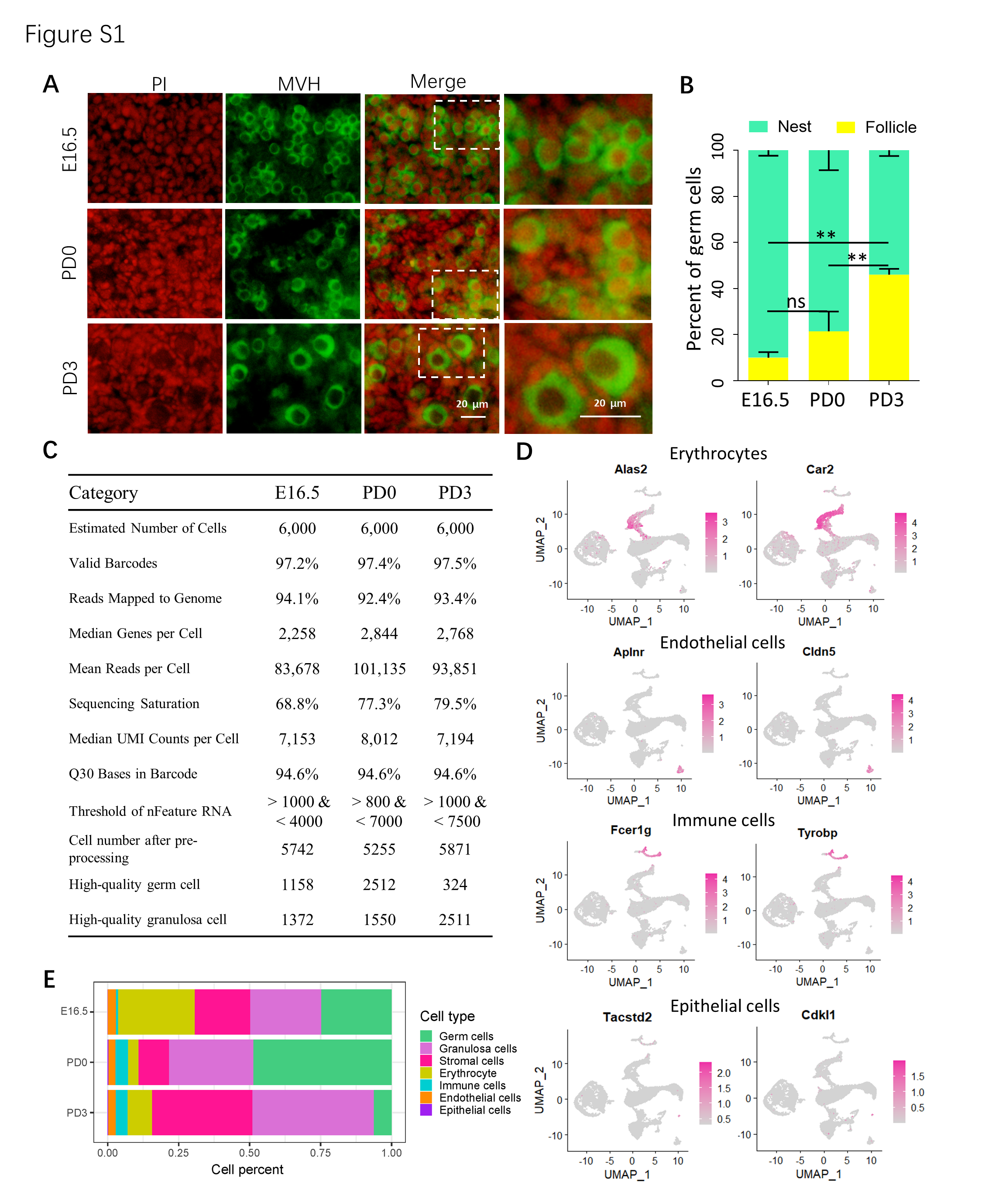

Supplement: S1 Fig — (A) Representative images of fetal ovaries at E16.5 and postnatal ovaries at PD0 and PD3. Germ cells were labeled with MVH (green) and nuclei counterstained with PI (red). Scale bar: 20 μm. (B) Percentage of germ cells in nests or follicles at the indicated stages. Data were represented with mean ± SD (n = 3 for independent repeats). The relative level was calculated between PD0 or PD3 and E16.5. Unpaired t tests are performed. Statistical significance is shown as ** P < 0.01; ns P >0.05. The raw data used for quantification of B can be found in S3 Data. (C) The sequenced detail information of 3 samples after CellRanger and Seurat workflow. (D) Feature plots of specific marker genes of erythrocytes, endothelial cells, immune cells, and epithelial cells. (E) Percentages of the 6 ovarian cell types at E16.5, PD0, and PD3. The sequencing data was deposited availably in GSE134339, and this figure can be produced using scripts at https://github.com/WangLab401/2020scRNA_murine_ovaries. E16.5, embryonic day 16.5; PD0, postnatal day 0; PD3, postnatal day 3; MVH, mouse vasa homologue; PI, prodium iodide. (TIF) [file pbio.3001025.s001.tif]

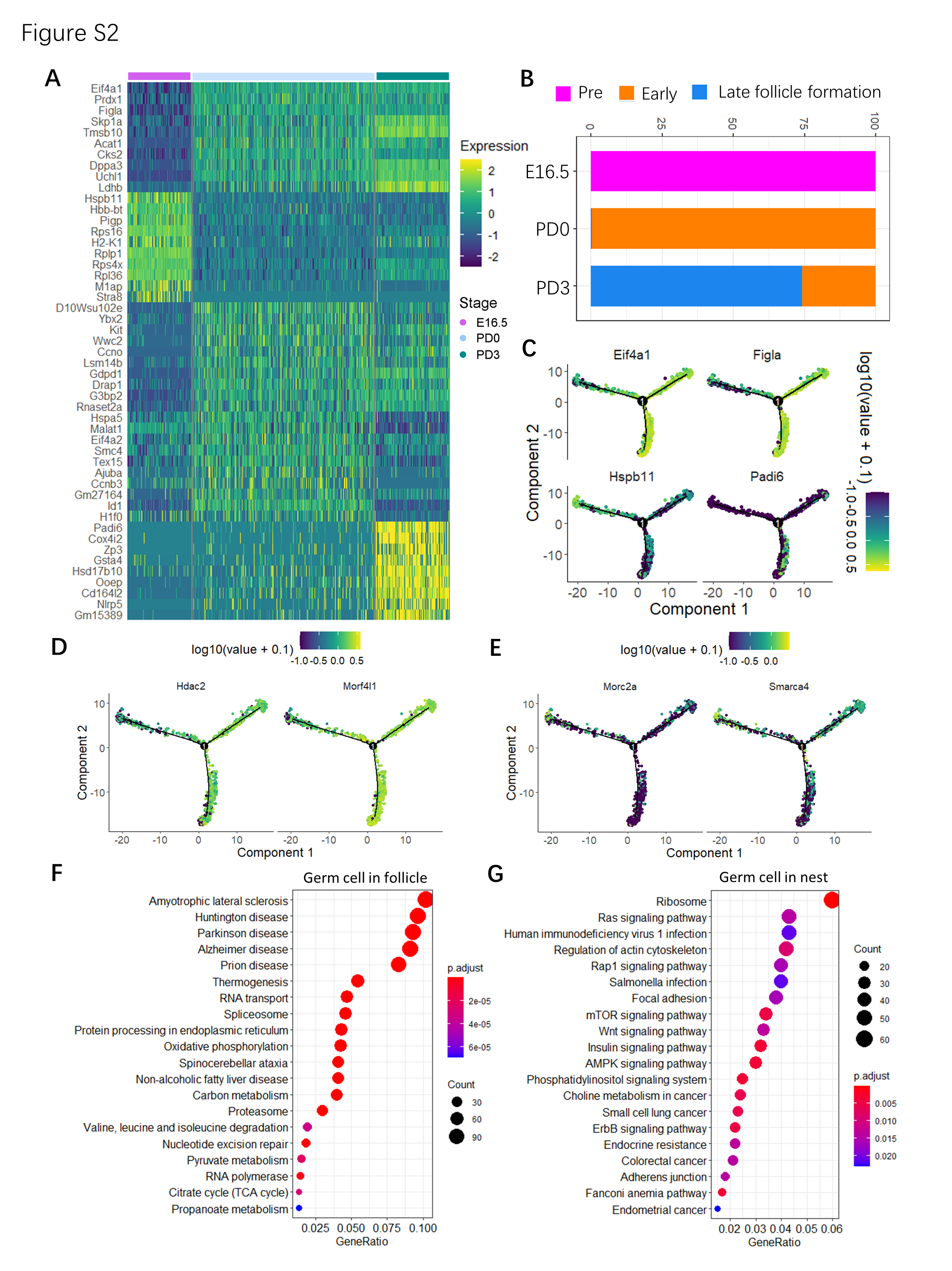

Supplement: S2 Fig — (A) Heatmap of top 10 marker genes of germ cell cluster with developmental timeline. Top 50 marker genes in each cluster are shown in S2 Table. (B) Percentage of germ cells at pre-, early- and late-follicle formation stages. (C) The expressions of representative genes for 3 identified stages along with pseudotime trajectories. (D) Expression of representative genes (Hdac2 and Morf4l1) in GO term of “histone deacetylation” along with pseudotime trajectories. (E) Expression of representative genes (Morc2a and Smarca4) in GO term of “regulation of gene expression and epigenetic” along with pseudotime trajectories. (F) Pathway enrichment of highly un-regulated genes of germ cells in follicle. (G) Pathway enrichment of most expressed genes of germ cells in nest. More pathways are shown in S5 Table. GO, Gene Ontology. (TIF) [file pbio.3001025.s002.tif]

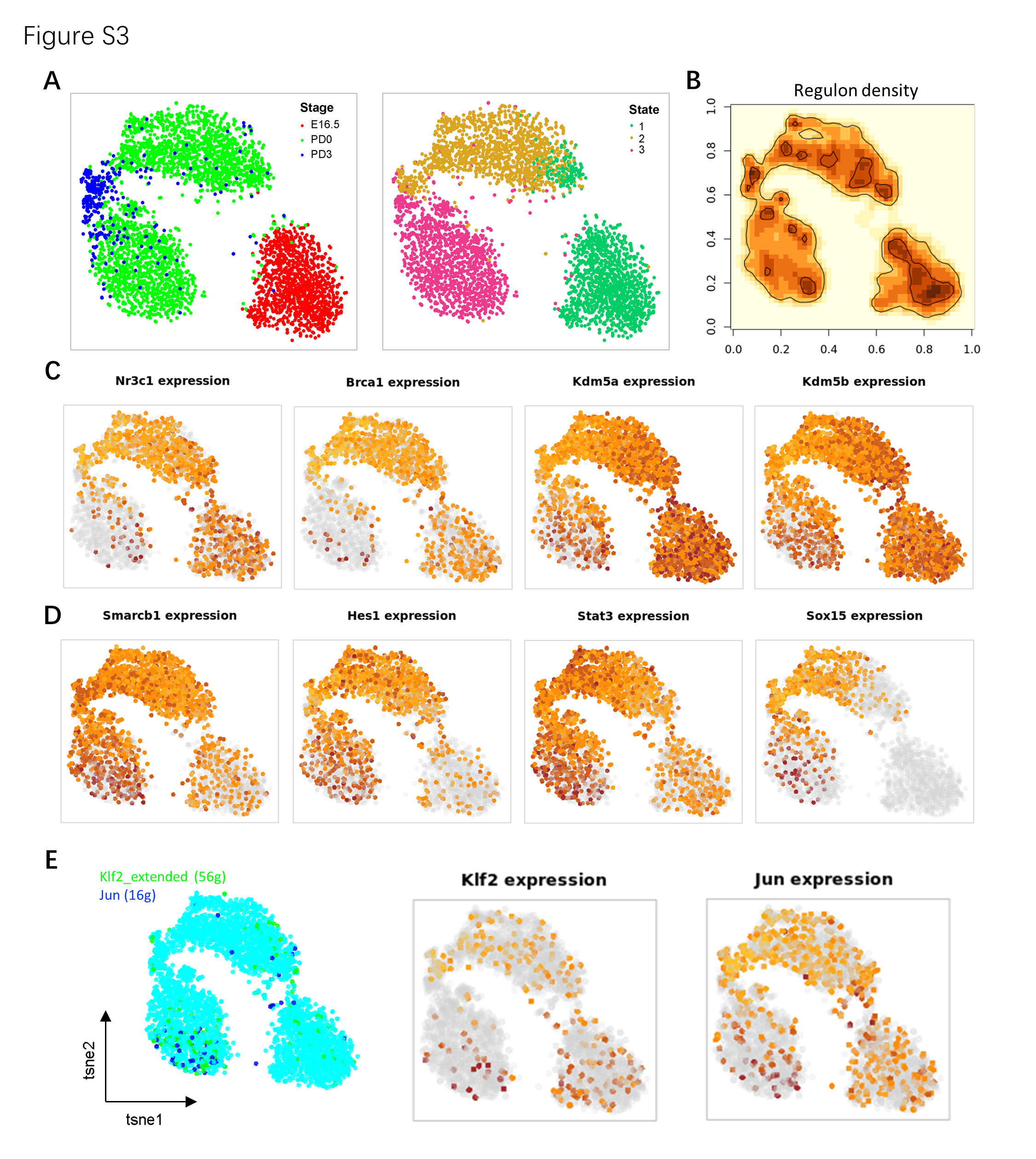

Supplement: S3 Fig — (A) t-SNE based on 201 regulons with 50 PCs and 50 perplexity according to the developmental stages (left) and cell states (right). (B) t-SNE of regulons activity density. Regulon density means “the occurrence frequency of regulon in cells.” (C) TF expressions of Nr3c1, Brca1 Kdm5a, and Kdm5b in selected cells. (D) TF expressions of Smarcb1, Hes1, Stat3, and Sox15 in selected cells. (E) t-SNE projection of average binary regulon activity (left) of Klf2 and Jun throughout the developmental stages and their expressions (right) in germ cells. TF, transcriptional factor; t-SNE, t-distributed stochastic neighbor embedding; PC, principal component. (TIF) [file pbio.3001025.s003.TIF]

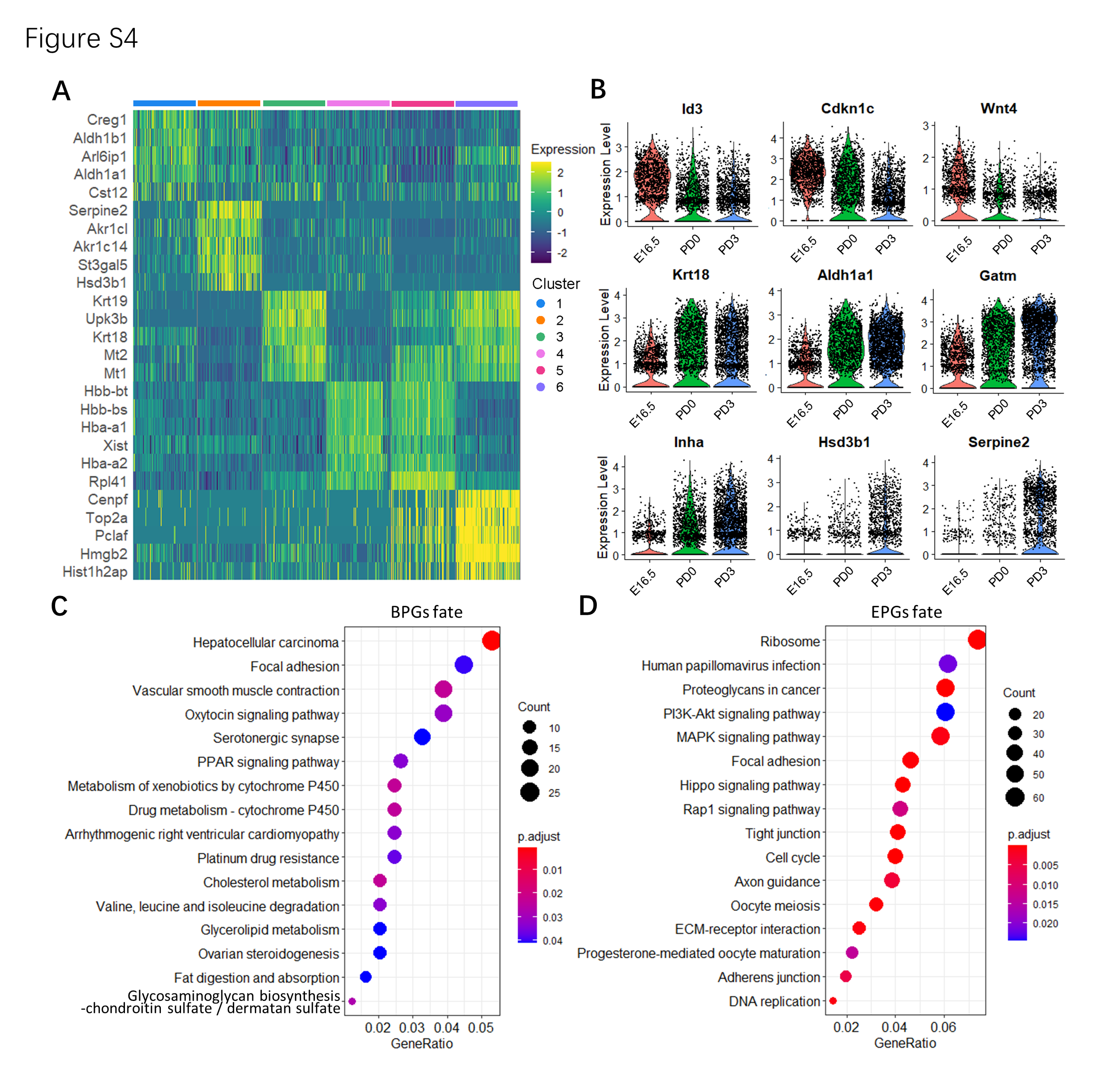

Supplement: S4 Fig — (A) Heatmap of the top 5 marker genes in granulosa cell clusters. Top 50 marker genes in each cluster are shown in S6 Table. (B) Vlnplots of the representative genes in granulosa cell clusters according to the developmental stages. (C) KEGG pathway enrichment of gene sets 1 and 2 that were related to BPGs fate. (D) KEGG pathway enrichment of gene sets 3 and 4 that have high expression in EPGs. More KEGG pathways are shown in S9 Table. The sequencing data was deposited availably in GSE134339, and this figure can be produced using scripts at https://github.com/WangLab401/2020scRNA_murine_ovaries. BPG, bipotential pre-granulosa; EPG, epithelial pre-granulosa; KEGG, Kyoto Encyclopedia of Genes and Genomes. (TIF) [file pbio.3001025.s004.TIF]

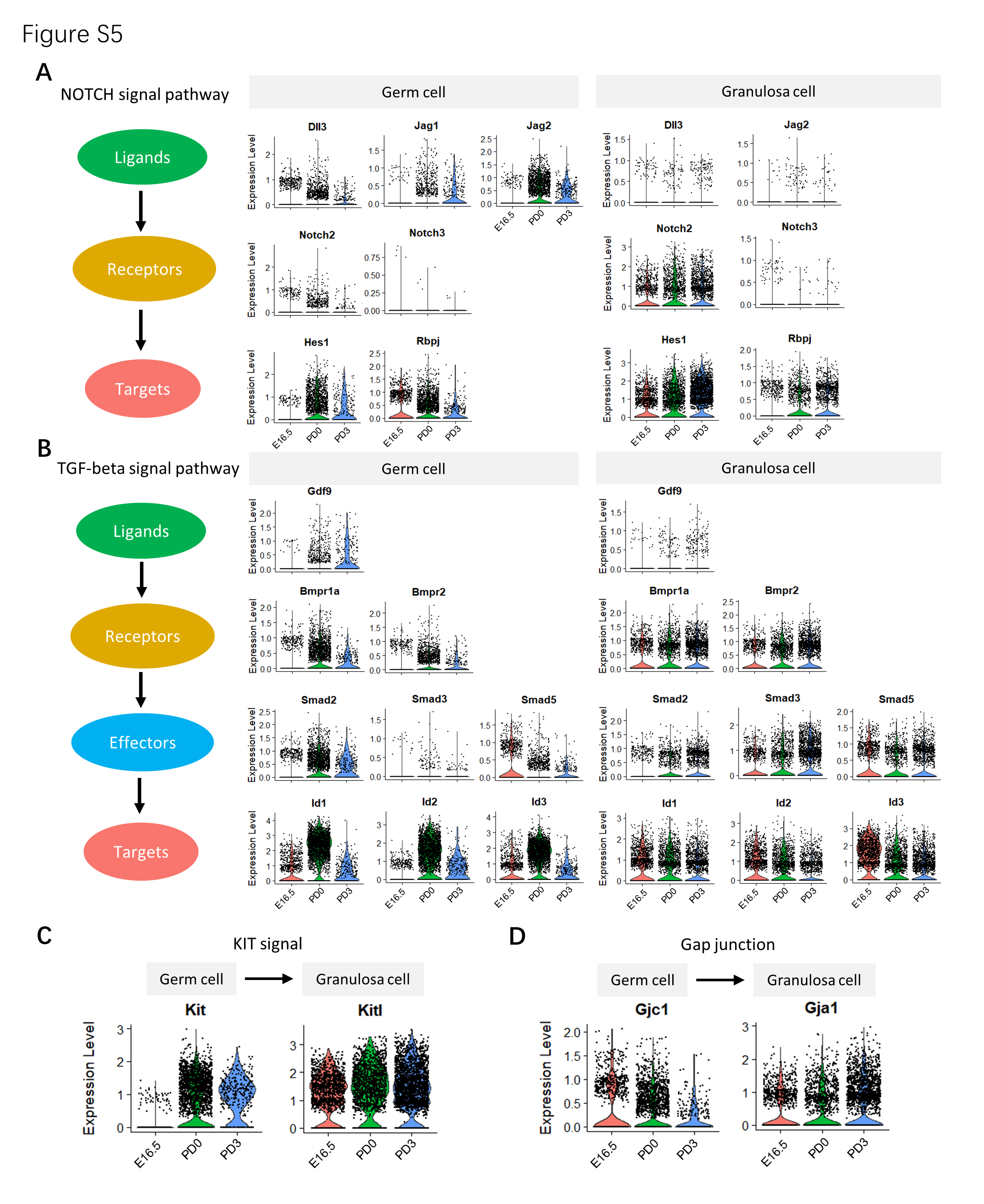

Supplement: S5 Fig — (A) Vnlplots of the expression of NOTCH signal ligands, receptors, and targets in germ cells and granulosa cells. (B) Vnlplots of the expression of TGF-beta signal ligands, receptors, effectors, and targets in germ cells and granulosa cells. (C) Vnlplots of the expression of Kit and Kitl in germ cells and granulosa cells. (D) Vnlplots of the expression of connexin genes of gap junction in germ cells and granulosa cells. TGF-beta, transforming growth factor beta. (TIF) [file pbio.3001025.s005.TIF]

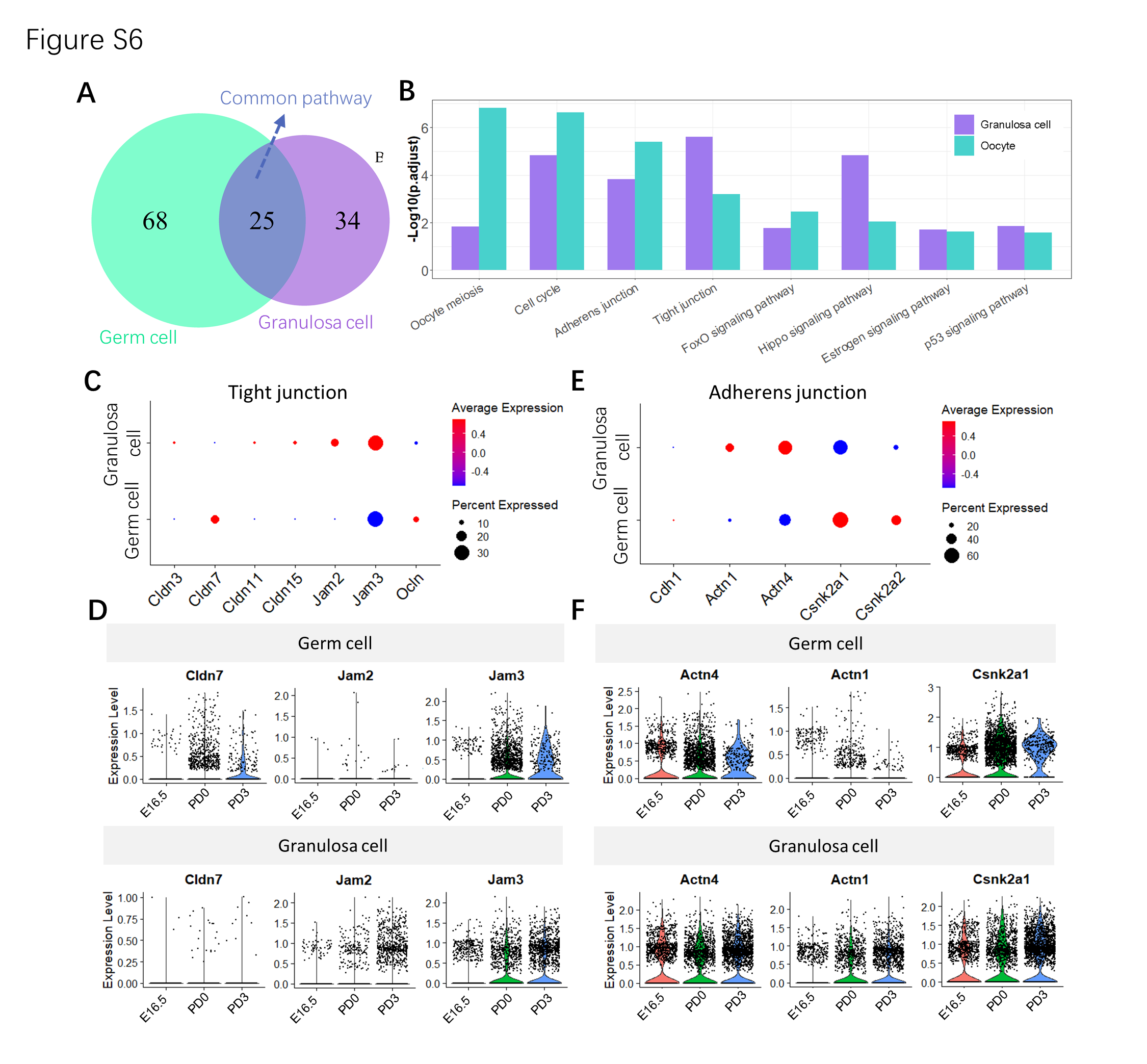

Supplement: S6 Fig — (A) Venn diagram of the common and specific pathway between germ cells and granulosa cells. (B) Histogram of the most representative common pathway of germ cells and granulosa cells. (C and D) Dot plots (C) and Vnlplots (D) of tight junction related genes in germ cells and granulosa cells. (E and F) Dot plots (E) and Vnlplots (F) of adherens junction-related genes in germ cells and granulosa cells. UMAP, uniform manifold approximation projection. (TIF) [file pbio.3001025.s006.tif]

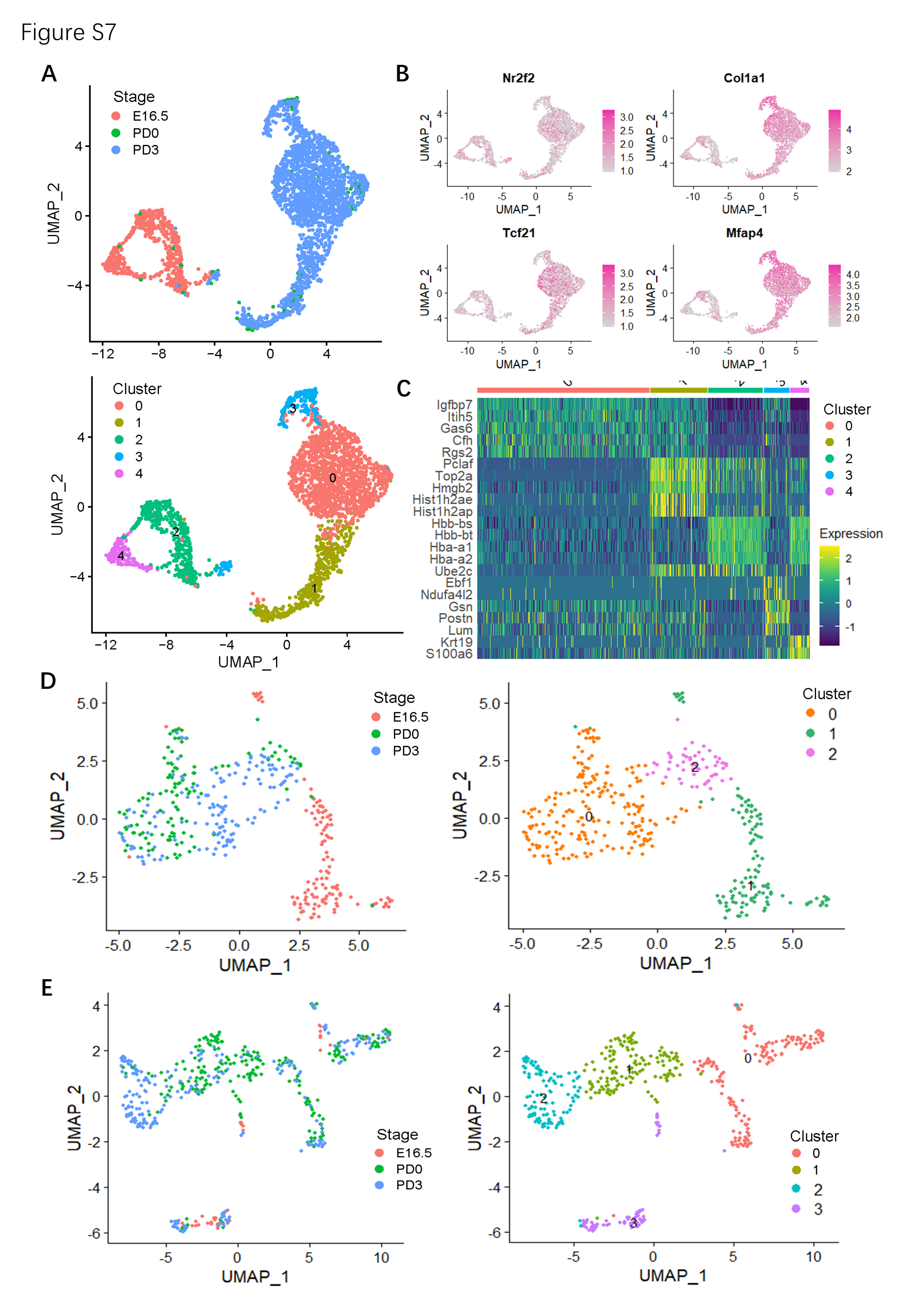

Supplement: S7 Fig — (A) Cluster analysis of stromal cells with UMAP plots based on developmental timeline (upper) and cell clusters (below). (B) Feature plots of known marker genes of stromal cells. (C) Heatmap of top 5 marker genes of stromal cell clusters. (D) Cluster analysis of endothelial cells with UMAP plots based on developmental timeline (left) and cell clusters (right). (E) Cluster analysis of immune cells with UMAP plots based on developmental timeline (left) and cell clusters (right). The sequencing data was deposited availably in GSE134339, this figure can be produced using scripts at https://github.com/WangLab401/2020scRNA_murine_ovaries. UMAP, uniform manifold approximation projection. (TIF) [file pbio.3001025.s007.TIF]

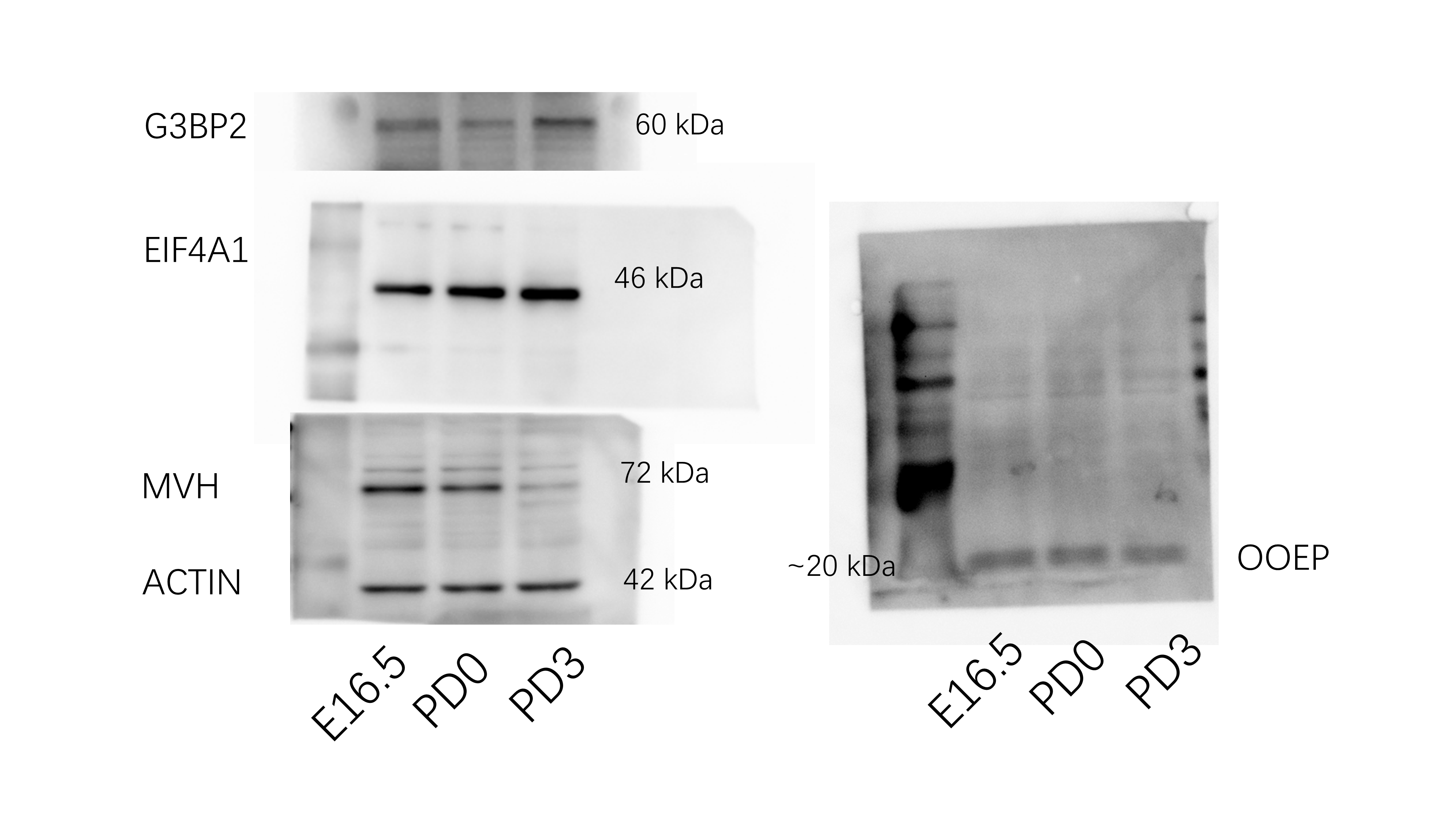

Supplement: S1 Data — (TIF) [file pbio.3001025.s018.tif]
