# Supplementary material for: Single-cell transcriptome landscape of ovarian cells during primordial follicle assembly in mice
Source: PLoS Biol. 2020 Dec 22;18(12):e3001025. doi: 10.1371/journal.pbio.3001025 (PMC7787681; doi:10.1371/journal.pbio.3001025)
Supplement: Attachment — Submitted filename: Reply to Reviewers.docx [file pbio.3001025.s021.docx]

**Reviewer #1:**

The manuscript by Jun-Jie Wang et al., profiled transcriptomes of fetal ovarian cells during primordial follicle formation by using single-cell RNA sequencing. The manuscript presented a significant amount of data that will serve as a very informative database for understanding many aspects of oocyte differentiation, granulose cell differentiation and primordial follicle assembly. The manuscript is well-written over all and the data are clearly presented. The following issues need be addressed by the authors.

**Answer:** Thanks for your evaluation. To explore the mechanisms of establishment for female ovarian reserve in the mouse, in this research, we performed single-cell sequencing using time-scenario ovary samples with embryo day 16.5, post-natal 0 and 3 day, which around the process of primordial follicle assembly. We expect to provide new insights in the biological process. Here, we are grateful for your comments, the best efforts would be made to improve our writing and figure data.

1. Abstract. Line 24. The timing of primordial follicle assembly is very different in mammals. It takes place perinatally in mice, but around mid-gestation in humans. Please correct this statement.

**Answer:** Your comment is important. We have made a revision. In the present study, the experimental animal object is mouse, so the writing “in mammals” of Abstract is replaced with “in the mouse”.

2. Introduction. Line 63, “ From here, PGCs move into….between E11.5-E12.5,”. The statement about the timing of PGC migration is not correct. Most PGCs arrive at the gonad by E10.5. (McLaren, 2003, Developmental Biology)

**Answer:** Thanks for your correction. We have corrected the improper statement, meanwhile, the carful check has been made to avoid a similar mistake. Thank you for your comment.

*“From here, PGCs begin to migrate, and most of them move into the embryo proper to initiate colonization by E10.5, between E11.5-12.5, the gonadal ridges form from the coelomic epithelium…”*

3. Introduction, Line 66-67. “The oocytes are closely associated in clusters, termed germ cell nests, in which they are connected to each other through cytoplasmic bridges formed…..during mitosis”. There is a fundamental difference between the term “germ cell nest” vs. “germline cyst” (Lei and Spradling, 2013, Development). “Germ cell nest” is used to describe the germ cells that are morphologically clustered. “Germline cyst” refers to sister germ cells that are actually connected by intercellular bridges. The term “oocyte nest” (line 88) does not exist.

**Answer:** Your comments are awfully important. The incorrect terms here have been revised. In addition, we also check the writing of “germ cell nest” vs. “germline cyst” in the whole manuscript to make our presentation more precise and scientific. Thanks for your professional correction.

4. Figure 2E, please explain the purpose of this graph? What does the changes in the percentage of cell types mean biologically?

**Answer:** We appreciate for your comments. Originally, the graph is presented to exhibit the cell type composition of the time-course ovaries sample in our study. The information here might be floating due to the preparation of single cell suspension and the random of capture in sequencing. As we have known, the number of germ cell is gradually decreasing from E16.5 to PD3 in the process of primordial follicle formation, but the number of E16.5 germ cell in our study is less than that of PD0 germ cells, it may arise from sample collection. At E16.5, most germ cells are connected to each other, in the digestion process of fetal ovaries for single cell solution, it’s likely that more germ cell still connected are discarded with the application of 40 μm filtrum, which mainly causes the underestimation of germ cell in number. And at PD3, the decreasing germ cells largely attribute to fetal oocyte attribution. Meanwhile, given the fact that the germ cell apoptosis peaks between PD1~PD3 in mice [1], the dying germ cells may be captured during the sequencing, these cells would be abandoned in the quality control of Seurat V3, it would further lead to the decline of germ cell in number. Thus, the present graph appears to be incompatible with the previous view, to avoid the misunderstanding, in the revised figures, we retained and moved the figure into supplementary figures.

**Reference:**

[1] Pepling M E, Spradling A C. Mouse ovarian germ cell cysts undergo programmed breakdown to form primordial follicles[J]. Developmental Biology, 2001, 234(2): 339-351.

5. Figure 3F, Why are the percentages of germ cells 25%-based? How was it calculated?

**Answer:** Thanks for your comments. The calculation of germ cell percentage is based on the ratio of germ cell number vs total cell number in each time point sample. According to our analyses, a genetic dynamic model, including pre-, early- and late-follicular formation stages were defined (Figures 3D and 3E), such as the marker genes expressed by germ cells at the pre- (i.e. M1ap, Hspb11, Pigp), early- (i.e. Eif4a1, G3bp2, and Acat1) and late- (i.e. Ooep, Padi6) follicle development stages were identified (Figures 3D and 3E). Moreover, the percentage changes of cells at the three stages from E16.5 to PD3 are shown in Figure 3F. In the revised figures, we moved Figure 3F into supplementary Figure S2C.

6. Figure 4G-I, How were the candidate genes chosen? Please explain it better in the result section. The same issue was found in Figure 5E, F.

**Answer:** We present the data to illustrate the cell fate, especially, the expression of genes enriched in GO term is displayed along with pseudotime trajectories. For example, Figure 4G and 4H are representative genes related to DNA methylation and chromatin changes (Figure 4G), also mitochondrial ATP synthesis (Figure 4H). Figure 4I showed the gene-related with adhesion. Figure 5E and 5F are genes enriched in PI3K-Akt signaling and apoptosis biological processes.

In the revised manuscript, we made a modification. In the pseudotime analysis of germ cell, the GO terms of “histone deacetylation” and “regulaiton of gene expression, epigenetic” were highlighted, and the representative genes “*Hdac2* and *Morf4l1*”, “*Morc2a* and *Smarca4*” enriched in the terms were respectively displayed along with pseudotime trajectories in Figures S2D and S2E.

7. Figure 5B, Figure legends are too small.

**Answer:** Thanks for your suggestion. We have revised the figure text in the revised figures.

8. Figure 6C. Please explain clusters better, in particular the cluster that is positive for Hes1 and Sox11. Based on the description in the results, Figure 6C is used to understand germ cell development. However, Gata4 and Maf are known transcription factors that are specifically expressed in ovarian somatic cells (De Falco et al., Developmental biology, 2011).

**Answer:** Thank you, the comments are very timely and nice. We revised our manuscript according your suggestion.

In the result of SCENIC, the regulatory network of transcriptional factors (TFs) and their target genes (term “regulons”) will be characterized. The TFs activity can be binarized into heatmap, and black blocks representing the TFs in cells that are ‘on’. Also, the projection map of TFs represents the cells population of their function, and the different TFs were marked with different colors. For example, the red color marks the Hes1 active (it means the Hes1 TF are “on”) cells, from the t-SNE and heatmap, the cells are mainly in State 1.

*“.., regulons activity was analyzed by AUCell (Area Under the Curve) software, where a default threshold was applied to binarize the specific regulons (“0” present “off” of TFs, and “1” refer to “on”). The parameters of t-SNE was set with 50 PCs (principal component) and 50 perplexity to visualize (after running a consistency test across several perplexity values and number of PCs),…” ---* ***see Method of “The regulon activity of transcription factors with SCENIC”***

*“(A)* *Heatmap of regulon activity analyzed by SCENIC with default thresholds for binarization. “On” indicates active regulons; “Off” indicates inactive regulons. The top rows represent the transcriptome signature of selected germ cells, they refer to the time-point label and State character of Monocle (left panel). The representative transcription factors and their motifs involved in follicle assembly were listed in the right panel.” ---****see Legend of “Fig. 5. Oocyte transcription factors involved in follicle assembly.”***

*“… Accordingly, regulon activity was binarized and matched with germ cell State and developmental stages (left panel), in addition, some representative regulons and their motifs (right panel) were listed (Fig 5A). As shown, a series of TFs displayed a more dynamic pattern. For example, Nr3c1, Brca1, Kdm5a and Kdm5b were active mainly at the germ cell nest stage (Fig 5B and S3C Fig), and Klf7 and Gabpa seemed to be mostly activated at the nest stage, but gradually turned off as follicular assembly occurred (Fig 5C). Similarly, Smarcb1, Hes1, Sox15 and Stat3 were mainly associated with States 2 and 3, corresponding to the period of primordial follicle assembly (Fig 5D and S3D Fig)….”* ***--- see Results of “Mapping oocyte-specific regulon networks by SCENIC”***

Moreover, for the issue of “Gata4 and Maf are known for ovarian somatic cells”, we have realized the problem, it might be derived from the doublets or multiplets of germ cells and somatic cells. In the revised manuscript, with stand pre-processing workflow of Seurat, the threshold value of unique feature counts (nFeature_RNA) was set strictly to abandon potential doublets or multiplets. In another, the germ cells specifically expressed marker gene of other cell types are discarded. With these methods, we obtain the high-quality captured cells for subsequent analyses.

In the revised results, we believe the results are more accurate. For instant, the TFs detected in germ cells within cyst in our present study, Nr3c1 has been revealed as the meiotic specific TF in oocyte, and Brca1, Kdm5a and Kdm5b are also detected in meiotic oocyte in previous report [1].

**Reference:**

[1] Ge W, Wang J J, Zhang R Q, et al. Dissecting the initiation of female meiosis in the mouse at single-cell resolution[J]. Cellular and Molecular Life Sciences, 2020 May: 1-19.

9. There are some minor English issues need to be fixed.

**Answer:** Thanks for your comment, we have checked and revised the English writing issues throughout the whole manuscript. In addition, the professional scholar devotes to checking and verifying the article.

**Reviewer #2:**

In their manuscript, “Transcriptome Landscape Reveals Underlying Mechanisms of Ovarian Cell Fate Differentiation and Primordial Follicle Assembly,” Wang et al perform single-cell RNA-Seq on mouse ovaries across a developmental series (E16.5, PND0, and PND3) to examine the changes in gene expression at the single cell level that accompany oocyte survival and death, granulosa cell differentiation, and crosstalk between oocyte-granulosa cell during primordial follicle assembly. Overall, this study is appears to be well done from a technical standpoint and harnesses the power of innovative new technology to provide further insight into a major developmental transition from germ cell cyst to primordial follicle. However, there are several important ways this study could be improved as outlined below:

**Answer:** We are grateful for your assessment. In the present study, the primordial follicle assembly process is focused through single-cell sequence with time-course early ovaries, and the big dataset will provide new underlying information, which is contribute for dissecting intrinsic mechanism of germ cell cyst breakdown and primordial follicle formation from single cell aspect. Here we have made the revision according to your comment.

1) A better rationale should be provided for why the specific mouse ages were used. For example, how do these ages correlate with the main biological events of GC invasion, cyst breakdown, and primordial follicle assembly.

**Answer:** The comment is very important. Here, the time-course ovaries, including embryo day 16.5 (E16.5), postnatal day 0 (PD0) and 3 (PD3), were selected for single-cell sequencing. As reported, female germ cells begin to enter the diplotene stage of first meiotic prophase at E16.5, subsequently, the pre-granulosa cells start to proliferated and invade germline cyst, separate the inter connected oocytes and form primordial follicles at E17.5. Most of cyst breakdown begins after birth, which is always followed by programmed cell death of oocyte, in a result, the survival oocyte is enclosed by a layer of pre-granulosa cells (pre-GCs) and primordial follicle finally begin to form [1-3]. Importantly, E16.5 time point represent the germ cells have get ready for the formation of primordial follicle (see prerequisites cellular events for the establishment of primordial follicles [4]), and PD0 is the initial time point of cyst breakdown. When it comes PD3, more primordial follicles have formed. The mouse ages used in the present study also are widely utilized in many professional publications of Reproduction studying the process of primordial formation [5-8].Finally, we have added the relative interpretation in our revised manuscript (showed in italic as bellow).

*“The selected three time points for sequencing represent the initial stage of a series of cellular events involving primordial follicle formation, i.e. cellular preparation, nest breakdown and primordial follicle assembly.”*

**Reference:**

[1] Pepling M E. From primordial germ cell to primordial follicle: mammalian female germ cell development[J]. Genesis, 2006, 44(12): 622-632.

[2] Pepling M E. Follicular assembly: mechanisms of action[J]. Reproduction, 2012, 143(2): 139-149.

[3] Wear H M, McPike M J, Watanabe K H. From primordial germ cells to primordial follicles: a review and visual representation of early ovarian development in mice[J]. Journal of Ovarian Research, 2016, 9(1): 36.

[4] Wang C, Zhou B, Xia G. Mechanisms controlling germline cyst breakdown and primordial follicle formation[J]. Cellular and Molecular Life Sciences, 2017, 74(14): 2547-2566.

[5] Niu W, Spradling A C. Two distinct pathways of pregranulosa cell differentiation support follicle formation in the mouse ovary[J]. Proceedings of the National Academy of Sciences USA, 2020, 117(33): 20015-20026.

[6] Huang K, Wang Y, Zhang T, et al. JAK signaling regulates germline cyst breakdown and primordial follicle formation in mice[J]. Biology Open, 2018, 7(1): bio029470.

[7] Jones R L, Pepling M E. KIT signaling regulates primordial follicle formation in the neonatal mouse ovary[J]. Developmental Biology, 2013, 382(1): 186-197.

[8] Zheng W, Zhang H, Gorre N, et al. Two classes of ovarian primordial follicles exhibit distinct developmental dynamics and physiological functions[J]. Human Molecular Genetics, 2014, 23(4): 920-928.

2) The title overstates the findings of this study. In fact, the authors focus primarily on known pathways. Discovery of known genes and pathways certainly validates this dataset, but does little to advance the field. Although this dataset will be a great resource for the field, a deeper analysis of this dataset should be performed with a focus on novel genes and pathways. The authors focus almost exclusively on the germ and somatic compartments of the ovary and overlook extrafollicular changes. The stromal compartment should be examined, especially given the rise in immune cells beginning at PND 0.

**Answer:** Thanks for your comments. After careful consideration, the title has been revised with “Single-cell Transcriptome Landscape of Ovarian Cells during Primordial Follicle Assembly in Mice”, which would be more pertinent and scientific.

In the present research, the single-cell transcriptome landscape of ovarian cells during primordial follicle assembly is displayed, which is mainly focused on the dataset, amounts of novel marker gene as well as pathways related to germ cell development and granulosa cell differentiation are revealed, and the preliminary verification is administrated as followed. For germ cells, the novel marker genes are examined with experiment, such as Hspb11 for pre-, G3bp2 and Eif4a1 for early-, and Ooep for late-follicle formation in germ cells. In term of pathway, it reveals that both TAZ, one core component of Hippo pathway, and FOXO3 proteins, an element of FoxO signal, expressed steadily, which suggest a potential involvement of Hippo and FoxO signal in the process of primordial follicle formation in the mouse.

In the revised manuscript, we have added the analysis of other somatic compartments of the ovary, including stromal cells, and endothelial cells immune cells. As shown in Figure S7A, the stromal cell line is clustering with developmental timeline (upper) and cell clusters (below) based on UMAP, Figure S7B shows the feature plots of specific marker genes of stromal cells, Figure S7C identified the novel marker genes in each cell cluster. Figure S7D and S7E are the cell clustering analysis of endothelial cells and immune cells, respectively.

*“In fact, some ovarian somatic cells, such as stromal (S7A-S7C, Figs) and endothelial cells (S7D Fig), showed different transcriptome patterns of pre- and post-natal development, and the immune cells were distinguished from them both (S7E Fig). However, due to the limited information available regarding other ovarian somatic compartments, the current study placed most attention on germ cells and granulosa cells.”*

3) Functional validation of novel pathways should be provided. For example, perturbation of pathways involved in key transitions should be performed to demonstrate biological significance.

**Answer:** Thank you very much, the comment is very vital and meaningful.

In the present data, to explore the novel pathway mediating the interaction of germ cells and granulosa cells, KEGG analysis is performed with both gene sets related germ cell and pre-granulosa cell development, the results showed the detailed molecular pathway involved in the fate decision at least one cell type, we also found part of overlap of the common pathway, which may mediate the interaction between the two cell populations. As we have known, some have not been reported to function during primordial follicle formation. For example, FoxO signal, specially FOXO3, is suggested to be connected to the establishment of dormant state in oocyte, however, the evidence of its role in primordial follicle formation is scarcely any. Moreover, the Hippo pathway is identified, together with another report that Lats1/2 maintaining the granulosa cell genetic program, both seem to suggest its role in granulosa cell differentiation. In addition, here we preliminarily verified the TAZ protein expression during primordial follicle formation with immunochemistry in time-course ovarian section.

The reviewer’s suggestion is very helpful, and the more detailed molecular regulatory mechanism would be detected in our future research.

4) This manuscript is written for an audience that is familiar with single cell sequencing technology and, therefore, contains a lot of jargon that makes it inaccessible to a general audience. Better explanations should be provided about terminology and methodology – e.g. clusters, pseudotime, regulon activity, SCENIC algorithm. Moreover, more information needs to be provided about how the methods reported here advance what has already been done in the field with respect to single cell sequencing.

**Answer:** The suggestion is great. We revised our manuscript, and the information and explanation of jargons for single-cell transcriptome analyses were added in revision.

The added contents were shown:

*“the multiple samples were performed an integrated analysis, briefly, the “FindIntegrationAnchors” function was used to identify anchors (a strategy for identification of cell pairwise correspondences between single cells across datasets, termed ‘‘anchors’’, which can be used to transform datasets into a shared space and construct the harmonized atlases at the tissue or organismal scale)”*

*“UMAP, a* *visualized method for cell clustering in high-dimensional transcriptomic data”*

*“…Further, the subpopulation of germ line or granulosa cell line was imported into Monocle (version 2.10.1) [82], a novel unsupervised algorithm, which re-ordered the cells to maximize the transcriptional similarity by their progress through differentiation, at the same time, it would distinguish genes activated early and later in differentiation, which contribute to dissecting of cell differentiation fate, also termed “pseudotime analysis””…”*

“*The single cell regulatory network inference and clustering (SCENIC) algorithm had been developed to assess the regulatory network analysis regard to transcription factors (TFs) and discover regulons (that is TFs and their target genes) in individual cells.*””

*“Finally, regulons activity was analyzed by AUCell (Area Under the Curve) software, where a default threshold was applied to binarize the specific regulons (“0” present “off” of TFs, and “1” refer to “on”).”*

*“Heatmap of regulon activity analyzed by SCENIC with default thresholds for binarization. “On” indicates active regulons; “Off” indicates inactive regulons. The top rows represent the transcriptome signature of selected germ cells, they refer to the time-point label and State character of Monocle (left panel). The representative transcription factors and their motifs involved in follicle assembly were listed in the right panel.”*

5) The authors should discuss the caveats and limitations of this technology and dataset.

**Answer:** Thanks for your comments. In recent years, single-cell RNA sequencing (scRNA-seq) has significantly advanced our knowledge of biological systems. This technology has been able to both study the cellular heterogeneity of organs and discover previously obscured cellular populations [1]. Presently, the single-cell sequencing is one of the most popular technology in life science field. The high throughput (thousands of cells in once sequencing) and low cost (for per cell on average) make it harboring enormous advantages. In the research, we applied it and discovered the dynamics gene signatures of germ cells and granulosa cells, which would contribute to dissect the novel regulatory mechanism of primordial follicle formation in the mouse. However, the limitation of this technology is not only the efficiency of cell capture (Chromium 10X with the most top about 65%) [2], but also restrict the cell number (~1000 cell/µL) and size (< 40 µm), which is not applicable for some precious samples. Meanwhile, the technology is not so kind for study regard to apoptosis. Accordingly, the non-viable and dying cells generally contain less RNA that is often more fragmented and may not be efficiently captured by 10X Genomics Single Cell Solutions [3,4].

**Reference:**

[1] Luecken M D, Theis F J. Current best practices in single‐cell RNA‐seq analysis: a tutorial[J]. Molecular Systems Biology, 2019, 15(6): e8746.

[2] AlJanahi A A, Danielsen M, Dunbar C E. An introduction to the analysis of single-cell RNA-sequencing data[J]. Molecular Therapy-Methods & Clinical Development, 2018, 10: 189-196.

[3] 10X Genomics Chromium Single Cell Gene Expression best practices.

[4] 10X Genomics^®^ Single Cell Protocols Cell Preparation Guide.

The added contents were shown:

*“… however, the present scRNA-seq platform does not excel in the determination of apoptotic cells, and dying cells are regarded as low-quality and therefore need to be discarded;…”*

*“Furthermore, despite of the fact that the single cell transcriptome technique is greatly expanding our insights into biological systems, especially regarding cellular heterogeneity, there are some unsolved issues that still need addressing within the field. Firstly, it’s the current lack of standardization for date analysis [75]. The problem is triggered by the growing number of analysis methods, exploding dataset sizes, and technical aspects (such as a variety of programming languages for data analysis tools), all of which prevent computational biologists from developing a gold standard practices. Another is regard to the incorporate of biological replicates [76, 77]. Although the biological replicates are suggested to eliminate batch effects, it seems the one replicate is still more popular in the single cell dataset of recent publications [47, 61]. In the present study, the application of one replicate may be considered as a potential limitation, however, the canonical knowledge in the field of primordial follicle formation, as well as the experimental verification, make us confident for our results. Moreover, the cell proportions studied here, at least partially, do not reflect the true cell type compositions in ovarian tissue (such as germ cells), this may be attributed to the methods of tissue handling and dissociation for single cell sample preparation, which is also present in another publication of single cell transcriptome investigating the female ovary [78].”*

6) There are minor grammatical issues throughout the manuscript which should be corrected.

**Answer:** We appreciate your comments. In the revised manuscript, much attention has been paid to the grammatical issues, we hope the new revision manuscript would make you satisfied. Thank you very much.

**Reviewer #3:**

Authors Jun-Jie Wang et al. address an unknown facet of oocyte and ovarian somatic cell interactions during germ cell cyst breakdown using single cell sequencing. In order to understand the cell-to-cell interaction in the developing ovary, the authors collected ovaries, in a time-course, at E16.5, P0, and P3 to include germ cell cyst breakdown and the germ cell-to-oocyte transition. In order to understand the role of individual cells during primordial follicle formation, the authors utilized single-cell sequencing to determine the dynamics of germ cells/oocytes and somatic cells/granulosa cells interactions. Included are numerous bioinformatics analyses. The authors determined that during this time course, germ cells were associated with ten distinct clusters and granulosa cells were associated with eight distinct clusters but were best described via pseudo-time and branching points. The interaction between the two cell types were determined by comparing transcriptomes. Overall, the authors corroborate patterns previously reported by numerous studies and have identified pathways that contribute to both germ cell cyst breakdown and the germ cell-to-oocyte transition. The strengths of this manuscript include the utilization of a novel method to extract a large amount of data on germ cells, somatic cells, and their interactions during germ cell cyst breakdown and germ cell-to-oocyte transition. The authors showed that some of their data are supported by other studies and that additional, unexplored pathways may be involved with primordial follicle assembly. Despite of its strengths, there are many major concerns, particularly on the details of the experiments, issues of single cell preparation, and the validity of the cell clusters. These concerns make it difficult to make proper interpretation and conclusion of the results. These concerns are:

**Answer:** Thanks a lot for your comments. In this study, the single-cell sequencing is applied to dissect the intrinsic mechanism of germ cell and pre-granulosa cell during primordial follicle formation. Here, the transcriptome landscape of ovarian cells is demonstrated, and part of experiment is designed to validate the dataset. In addition, we noticed there is amount of valuable information in data analysis, which is worth an in-depth study in the future. Thanks for your comments, we would make careful modifications accordingly and improve our research to be more scientific.

1.	Figure 1A&B: First, these data have been reported by many others so no new information is provided that warrant the inclusion of such data. Second, information on how these follicle and cyst numbers were obtained was not provided in Materials and Methods or anywhere in the manuscript. Third, it is not known where these numbers are consistent with the existing data in the literature.

**Answer:** Thank you for your nice comment.

For the first question, the reviewer is right and we have moved Figure 1A&B into the supplementary figure and adjusted our Figure 1 accordingly.

For the second one, we have carefully revised the Materials and Methods part regarding single cell prepration as the reviewer suggested, please refer to our revised manuscript.

*“In addition, the germ cells with more than two cells inter-connected were considered as nest germ cells in ovarian sections, whereas single germ cell without connection to each other were considered as follicle germ cells; the percentage of germ cells within nests or follicle was calculated.”*

For the third one, we admit that there are some differences between our dataset here and previously published reports. The reasons were: Firstly, the biological event of oocyte attrition after birth should be the main reason for the decrease of oocyte numbers. As reported, large number of oocytes are lost as they assemble to form primordial follicles in the first days after birth, as a result, approximately 6.4 germ cells on average in each cyst survive from the initial 30 (20%) by PD4 in the mouse as primary oocytes [1,2]. Another, given the fact that the germ cell apoptosis peaks PD1~PD3 in mice [3], the dying germ cells may be captured during the sequencing, these cells would be abandoned in the quality control process, it would further lead to the decline of germ cell in number for PD3 stage. These reasons together may responsible for the differences observed in the current study.

**Reference:**

[1] Wang C, Zhou B, Xia G. Mechanisms controlling germline cyst breakdown and primordial follicle formation[J]. Cellular and Molecular Life Sciences, 2017, 74(14): 2547-2566.

[2] Kerr J B, Myers M, Anderson R A. The dynamics of the primordial follicle reserve[J]. Reproduction, 2013, 146(6): R205-R215.

[3] Pepling M E, Spradling A C. Mouse ovarian germ cell cysts undergo programmed breakdown to form primordial follicles[J]. Developmental Biology, 2001, 234(2): 339-351.

2.	Figure 1D: How many ovaries were used for each developmental stage? There are more clusters from E16.5 ovary and much less in other two stages. It is possible that more cells at E16.5 increases the granularity for this particular stage. This poses a major issue on the potential bias on clustering and the biological meaning of such clustering.

**Answer:** Thank you for pointing this out. For each developmental stage, about 4-6 ovaries were collected for postnatal ovarian samples, and E16.5 sample was derived from the fetal ovaries of two mothers in pregnancy. We have added these information in the Materials and Methods accordingly (revised Figure S1C). For the second question, to avoid the potential bias, in our revised manuscript, the same number of cells (6000) were randomly chosen to reduce potential bias on clustering for subsequent analysis by the parameter of “--force-cells” in the CellRanger. These information have been added in our revised manuscript, please refer to Figure S1C.

3.	The information on single cell analysis lacks details. For example, what was the recovery rate of the cells? What were the average barcodes, reads, and genes for each single cell? How many replicates were included? What was the sequencing depth, recovery rate for the cells, and duplets/multiplets? When the single cell sequencing technique started a while ago, it may be acceptable to have only a single sample in the past but now the field has realized that at least a duplicate is necessary. It is unacceptable with just a single sample for each time point.

**Answer:** Thank you very much for your comments.

For the first three questions, we have attached a table describing sequencing parameters in our revised manuscript, please refer to Figure S1C (or see bellow). For the removal of duplets/multiplets, we utilized strigent quality control methods to minimize its effects. First, in quality control of Seurat, the cell with over high unique feature counts (likely duplets/multiplets) are filtered. At the same time, in the population of single cell type, the cell specifically expressed another cell type maker gene are discarded. For example, in subpopulation of germ cell, the cell cluster specifically expressed granulosa cell, stromal cells, or other cell type marker genes are throwed. Moreover, the abnormal high expression may be duplets/multiplets.

| Category | E16.5 | PD0 | PD3 |
| --- | --- | --- | --- |
| Estimated Number of Cells | 6,000 | 6,000 | 6,000 |
| Valid Barcodes | 97.2% | 97.4% | 97.5% |
| Reads Mapped to Genome | 94.1% | 92.4% | 93.4% |
| Median Genes per Cell | 2,258 | 2,844 | 2,768 |
| Mean Reads per Cell | 83,678 | 101,135 | 93,851 |
| Sequencing Saturation | 68.8% | 77.3% | 79.5% |
| Median UMI Counts per Cell | 7,153 | 8,012 | 7,194 |
| Q30 Bases in Barcode | 94.6% | 94.6% | 94.6% |
| Threshold of nFeature RNA | > 1000 &  < 4000 | > 800 &  < 7000 | > 1000 &  < 7500 |
| Cell number after pre-processing | 5742 | 5255 | 5871 |
| High-quality germ cell | 1158 | 2512 | 324 |
| High-quality granulosa cell | 1372 | 1550 | 2511 |

For the fourth question, the matter of biological replicates is still an open question, and thanks a lot for the reviewer’s suggestion. According to “Experimental planning guide-Getting started with single cell gene expression”, it states “in some studies, one sample alone can be seen as sufficient, where each cell represents a biological replicate and different samples from different individuals account for the variability of a particular biological process …”. Moreover, the biological replicates are unfeasible due to the enormous cost of library preparation and additional sequencing. Given that the statistical significance for calculation of gene expression, each cell within single-cell sample can be considered a single replicate [1]. In another, the single sample for each time point is still frequent in the most published reports [2-4].

**Reference:**

[1] Wu A R, Neff N F, Kalisky T, et al. Quantitative assessment of single-cell RNA-sequencing methods[J]. Nature Methods, 2014, 11(1): 41.

[2] Niu W, Spradling A C. Two distinct pathways of pregranulosa cell differentiation support follicle formation in the mouse ovary[J]. Proceedings of the National Academy of Sciences USA, 2020, 117(33): 20015-20026.

[3] Kalucka J, de Rooij L P M H, Goveia J, et al. Single-cell transcriptome atlas of murine endothelial cells[J]. Cell, 2020, 180(4): 764-779. e20.

[4] Adams T S, Schupp J C, Poli S, et al. Single-cell RNA-seq reveals ectopic and aberrant lung-resident cell populations in idiopathic pulmonary fibrosis[J]. Science Advances, 2020, 6(28): eaba1983.

4.	tSNE used to be the only way for clustering but its reproducibility has become an issue. The field has moved away from tSNE and now uses UMAP for most analyses. I strongly urge the authors to reanalyze all of their data using UMAP. The final results could be quite different from what they have now, particularly in regard to the statistical power that define individual clusters.

**Answer:** Thank the reviewer very much for the helpful suggestion. As the reviewer suggested, we reanalyzed our dataset with Seurat V3 pipeline, and the UMAP has also been utilized in our revised manuscript. Indeed, the method distinguishes the different cell types apparently, the same cell population is clustering more closer in space. We appreciate your comment very much.

5.	Figure 2: There are issues/discrepancy on the clusters/definition of what cell populations they represent and what is already known about the ovary at these stages. Where are the interstitial/stromal cell populations? Interstitial cells represent a significant portion of the cells composing the fetal ovary, and certainly more cells than epithelial cells or erythrocytes. Clusters for interstitial cells should therefore show up at all stages, with an increase in number of cells overtime. For instance, genes such as Nr2f2 (COUPTFII), Acta2 (aSMA), or Tcf21 should be specific / strongly enriched in stromal cell populations. However, these genes were found in granulosa cells (cluster 12), epithelium (clusters 0, 9, 13 for Acta2 and clusters 9 and 12 for Tcf21). This raises the concerns on the single cell preparation and the quality of the cells.

**Answer:** Thank the reviewer for pointing this out. We noticed the issue here, as reported, after the formation of the gonadal ridge and sex differentiation, the stroma from the mesonephros penetrates further into the gonadal ridge/ovarian primordium, which creates areas of stroma, characterized by expression of NR2F2 in stromal cells, and produces the ovigerous cords [1,2]. In our revised manuscript, we used Tcf21 and Nr2f2 genes to label stromal cells, please refer to Figures 1C and 1E. Meanwhile, the stromal cell population also is extracted to performat cell clustering, and some novel marker genes are identified (Figures S7A, S7B and S7D). The comment is great, we do appreciate it.

**Reference:**

[1] Hummitzsch K, Irving-Rodgers H F, Schwartz J, et al. Development of the mammalian ovary and follicles[M]//The ovary. Academic Press, 2019: 71-82.

[2] Hummitzsch K, Anderson R A, Wilhelm D, et al. Stem cells, progenitor cells, and lineage decisions in the ovary[J]. Endocrine Reviews, 2015, 36(1): 65-91.

6.	Similarly, the clusters labelled “epithelial cells” represent a high number of cells, up to 30% of all cells at P3 based on Figure 2E. This is not physiologically representative of the population size of epithelial cells, which should correspond to a much smaller cluster.

**Answer:** Thank the reviewer for your comments. In our revised manuscript, we used more stringent markers to characterize different cell populations, and the epithelial cells were consisted of a very small number of cells in the ovary at this age. Please refer to our revised Figure 1D.

7.	Figure 2: It is not scientifically sound to use one single sample of single cell clusters to define the percentage of various cell types over time. The case in point is germ cells: how is it possible that the percentage of germ cells at PD0 is more than 50% whereas 20% at E16.5?

**Answer:** Thank the reviewer for pointing this out. The reviewer is right. In the current study, cell percentage of various cell types over time is not feasible due to the existence of germ cell cyst, and fetal oocyte attrition, which makes it hard to reveal the in vivo scenario when cells were dissociated into single cells (detailed see comment 8), we therefore move it in Figure S2E.

8.	It is surprising that the % of oocytes / total cells increases from E16.5 to P0, suggesting that the number of oocytes increases during this period, it goes against all previously published quantifications (Lei Lei 2013 ; Grive et al., 2014; Malki et al., 2014 and others). How can the number of oocytes increase when they have already entered meiosis? Could it be a technical problem of dissociation / capture of germ cell populations at E16.5 that skew the % cell distribution? At E16.5, most germ cells are still in nests and physically connected to each other through intercellular bridges. Could it be possible that some elements captured during the single cell experiment at E16.5 and considered as one single germ cell could actually corresponds to several germ cells still connected, and therefore resulting in under-estimating the germ cell % at E16.5?

**Answer:** Thank the reviewer for the comment.

Originally, the number of germ cells is decreasing followed the process of primordial follicle formation. For the initial stage of E16.5, the germ cell number should be at the top for count among the time-course samples. Here, the germ cell percent at E16.5 seems to be underestimated, the reason causing the phenomenon is suggested to be the ovarian digestive efficiency. As we known, most germ cells are physically connected to each other at E16.5, which may lead to inefficient digestion for fetal ovaries, and more germ cell still connected are discarded in filtration, which mainly causes the underestimation of germ cell in number.

Moreover, in the preparation of the sample for sequencing, after digestion, the filtrum of 40 μm is implemented, and the observation at counting instrument is checked to guarantee the single cell suspension, which largely avoids the doublet/multilets, it may reject more germ cells still connected, and also give rise the underestimation of germ cell number at E16.5 stage.

9.	Figure 3: There were so few cells for P3. Even though oocytes go through attrition, there should not be such a huge difference in number of cells when compared to P0. It is possible that so few cells for P3 stage compared to other stages affect the clustering of the germ cells and prevent seeing the heterogeneity present at this stage.

**Answer:** Thanks, your comments are meaningful. For the low number of germ cell at PD3 in our study, it’s believed to be multicausative. Firstly, the biological event of oocyte attrition after birth should be the main reason for the decrease of oocyte numbers. As reported, large number of oocytes are lost as they assemble to form primordial follicles in the first days after birth, as a result, approximately 6.4 germ cells on average in each cyst survive from the initial 30 (20%) by 4 post-natal days in the mouse as primary oocytes [1,2]. Another, given the fact that the germ cell apoptosis peaks between PD1~PD3 in mice[3], the dying germ cells may be captured during the sequencing, these cells would be abandoned in the quality control process, it would further lead to the decline of germ cell in number for PD3 stage. Thus, we suggest the low number of germ cell is acceptable.

Moreover, despite only a small population of germ cell at PD3 compare to PD0, we still have obtained more than 300 germ cells with high-quality (see supplementary Figure 1C), which present special gene expression pattern. Also, we have found that the inconsistent number of cells in single cell analysis is pervasive and seems to be tolerable [4,5].

**Reference:**

[1] Wang C, Zhou B, Xia G. Mechanisms controlling germline cyst breakdown and primordial follicle formation[J]. Cellular and Molecular Life Sciences, 2017, 74(14): 2547-2566.

[2] Kerr J B, Myers M, Anderson R A. The dynamics of the primordial follicle reserve[J]. Reproduction, 2013, 146(6): R205-R215.

[3] Pepling M E, Spradling A C. Mouse ovarian germ cell cysts undergo programmed breakdown to form primordial follicles[J]. Developmental Biology, 2001, 234(2): 339-351.

[4] Niu W, Spradling A C. Two distinct pathways of pregranulosa cell differentiation support follicle formation in the mouse ovary[J]. Proceedings of the National Academy of Sciences USA, 2020, 117(33): 20015-20026.

[5] Sohni A, Tan K, Song H W, et al. The neonatal and adult human testis defined at the single-cell level[J]. Cell Reports, 2019, 26(6): 1501-1517. e4.

10.	Fig. 3C: Clusters on the heat map could be organized per time-point in order to more easily associate the genes listed to the age of the ovary.

**Answer:** The suggestion is good. In the revised figures, we also displayed the heat map of cluster marker gene by time-point manner in Figure S2B. It showed the perfect consistency with the divided stage, that is pre-, early- and late- primordial follicle formation.

11.	Figure 4, 5 and 6: Have the authors confirmed the expression profile of some of the genes using in situ hybridization or immunostaining? All sorts of projections can be generated from single cell data. But it is also glaringly questionable how much biological relevance these projections can actually predict.

**Answer:** The suggestion is very good. As we have known, the process of primordial follicle formation involves amounts of biological events. By pseudotime analysis (Figure 4 and 5), each cell is ordered along with the developmental trajectories, and the molecular signature related to cell fate transition is revealed. Not only the present cognition for follicular assembly is verified with the evidence of data set, but also the novel insights that potentially involve in cyst breakdown and follicle are revealed from single cell perspective. In addition, Figure 6 demonstrated the mapped transcriptional factors (TFs) for different oocyte fates. Specificly, the TFs detected in germ cells within cysts in our present study, Nr3c1 and Brca1, Kdm5a and Kdm5b are also detected in meiotic stage oocyte in previous report [1].

Meanwhile, to elevate the reliability of our study, we performed the immunochemistry for verify the data set results. For the novel marker genes of germ cells, with experiment, such as Hspb11 for pre-, G3bp2 and Eif4a1 for early-, and Ooep for late-follicle formation in germ cells are examined. In terms of the pathway, it reveals that both TAZ, one core component of the Hippo pathway, and FOXO3 proteins, an element of FoxO signal, expressed steadily. The experimental results are all consistent with our data analysis.

**Reference:**

[1] Ge W, Wang J J, Zhang R Q, et al. Dissecting the initiation of female meiosis in the mouse at single-cell resolution[J]. Cellular and Molecular Life Sciences, 2020 May: 1-19.

12.	Please define “regulon” and “regulon density” in Figure 6. These are not terms known by many readers.

**Answer:** Thanks for your comments. The description of terms like this in data analysis had been added in Method. In SCENIC, “Regulon” means “The small gene regulatory network consisting of a transcription factor connected to its direct target genes”. In its workflow, with the count matrix depicting the gene abundances for all cells as input, GENIE3 module is applied to identify putative direct-binding targets, and each co-expression module is subjected to cis-regulatory motif analysis using RcisTarget, only modules with significant motif enrichment of the correct upstream regulator are retained, which is suggested to be “regulons”. And “regulon density” refers to “the frequency of occurrence for regulons in cells”. We have added the relative interpretation in the revised manuscript.

*“…Regulon density means “the occurrence frequency of regulon in cells”…”*

13.	Figure 6A: It is almost impossible (at least to me) to understand what this figure means. The four rows are the top of the figure were never explained.

**Answer:** Thanks for your comments. In Figure 6A and 6B, it showed the SCENIC clustering of dimensionality reduction for germ cells with Seurat and Monocle label, which would contribute to understand the cell status. And for Figure 6C, the binary activity for each cell is generated from the SCENIC AUC distribution and plotted as a heat map, with black blocks representing TFs that are ‘on’ in cells. the top rows represent the transcriptome signature of selected germ cells, which refer to the number of expressed gene, number of UMI, time-point label and Monocle character.

In our revised manuscript, we have added these information:

*“Accordingly, regulon activity was binarized and matched with germ cell State and developmental stages (left panel), in addition, some representative regulons and their motifs (right panel) were listed (Fig 5A).”*

14.	Fig 6C: purple cluster contains many TF not expressed in the oocytes and are specific of somatic cells. Ex: GATA4, WT1, EMX2. This raises the possible contamination and also multiplets (multiple cells in one droplet).

**Answer:** Thank the reviewer very much for the nice comment. The reviewer is right, and in our revised manuscript, we reanalyzed our dataset with a more stringent quality control standard (see revised materials and methods part for details) using Seurat V3’s UMAP function. Followed the method, the strict quality control has been carried out to avoid the multiplets. First, the distribution of nFeature_RNA is checked to abandon the cells with over number expression (potential doublets or multiplets), and the germ cells expressed marker genes of other cell types are discarded. All these measures are arranged to decontaminate the contamination. And the TFs results are accordingly revised. Thanks for your kindly remind.

In the revision, we believe the results are more accurate. For instant, the TFs detected in germ cells within cysts in our present study, Nr3c1 has been revealed as the meiotic specific TF in oocyte, and Brca1, Kdm5a and Kdm5b are also detected in meiotic oocyte in the previous report [1].

**Reference:**

[1] Ge W, Wang J J, Zhang R Q, et al. Dissecting the initiation of female meiosis in the mouse at single-cell resolution[J]. Cellular and Molecular Life Sciences, 2020 May: 1-19.

15.	More explanation and description should be provided on how “granulosa cell” clusters were selected in Figure 7A for further analyses.

**Answer:** Thanks for your comments. We revised the manuscript to supplement the detailed information. In fact, the cells strongly expressed common granulosa cell marker genes are defined as granulosa cell (showed in Figure 2, or newly revised Figure 1D), such as *Amhr2, Fst, Foxl2,* and *Kitl* gene.

16.	The interpretation of the pseudo time analyses for the granulosa cells is questionable. The pre-granulosa cells present at E16.5 (state 1 in Fig.7E) do not give rise to pre-granulosa cells expressing Lgr5/Rspo1. This has been shown in multiple publications. Lgr5+ pre-granulosa cells arise from the ovarian surface epithelium, which is a different cell population than the E16.5 pre-granulosa cells expressing Foxl2 / Cdkn1c. So basically, the pseudo time analyses are performed with cell populations of different sources resulting in bias in the bioinformatic analysis.

**Answer:** Thanks for your comments. According to the suggestion of reviewers, we apply Seurat V3 to reanalysis our data. In the revision, we improve our analysis by changing the ordering gene with differentially expressed genes (>6000) of cell clusters with Monocle, which represent more transcript signatures of selected cells and make results more accurate, not high variable genes (2000) identified by Seurat (previous). In the revised figures, we provide the information of *Lgr5* and *Foxl2* expression.

For example, most cells at State 1 are at E16.5, and strongly express *Foxl2* gene, so it is believed to be early cyst stage pre-granulosa cells; as development proceed, the differentiated pre-granulosa cells express *Lgr5*; at last stage, granulosa cells are labeled with high expression of *Hsd3b1*, which are required for steroidogenesis. Also, the dynamics of gene signature in developmental granulosa cells is consistent with other previous reports [1,2]. In addition, the results suggsted State 3 cells with high expression of Foxl2, Aard and Akr1c14 were suggested to be bipotential-derived pre-granulosa cells (BPGs); the State 2 pre-granulosa cells (EPGs) were originated from ovarian surface epithelium, evidenced by Lgr5, Gng13 and Krt19 expression, and both of them are resulted with granulosa cells of two waves follicle formation in ovarian cortex (EPGs) and medulla (BPGs).

**Reference:**

[1] Stévant I, Kühne F, Greenfield A, Chaboissier MC, Dermitzakis ET, Nef S. Dissecting Cell Lineage Specification and Sex Fate Determination in Gonadal Somatic Cells Using Single-Cell Transcriptomics. Cell Rep. 2019 Mar 19;26(12):3272-3283.e3.

[2] Niu W, Spradling A C. Two distinct pathways of pregranulosa cell differentiation support follicle formation in the mouse ovary[J]. Proceedings of the National Academy of Sciences USA, 2020, 117(33): 20015-20026.

17.	Figure 8E&G: Has any of these expression pattern been confirmed by in situ hybridization or immunostaining?

**Answer:** Thanks for your comments. In the revised figures, we carried out the experiment to confirm the expression of key proteins, including TAZ in Hippo and FOXO3 of FoxO signal for germ cells.

*“Moreover, the expressions of FOXO3 and TAZ in germ cells were confirmed at the protein level by immunohistochemical staining (Fig 7I), they were mainly expressed at the postnatal stage, which verified the data analysis results.”*

18.	One of the main purposes of single cell sequencing is to identify novel cell types and pathways. Most of the candidate genes or pathways found in the analysis were previously described. What are the new information/mechanism the derive from this study?

**Answer:** Thank the reviewer very much for the comment, as the reviewer suggested, in our revised manuscript, we indeed identify amount of novel marker genes in germ cells and granulosa cells. In particular, the novel marker genes in germ cells have been verified via immunohistochemistry staining with ovarian sections (For example, Hspb11 for pre-, G3bp2 and Eif4a1 for early-, and Ooep for late-follicle formation in germ cells were examined by immunohistochemistry or western blot). Also, the stage-specific pathway for germ cell and granulosa cell is revealed. For example, some novel signals, such as the Hippo signal may involve in follicle fate of germ cell, and mTOR and Wnt for cyst stage germ cells (revised Figure S2D and S2E). Further, the common pathways are highlighted in our research, which may involve in the interaction of germ cells and granulosa cells. Actually, some novel mechanism regard to alternative of germ cells or granulosa cells are displayed in Figure 8A. Such as, Hippo and FOXO signal pathway are revealed to be involved in primordial follicle formation, which has not been examined previously. In addition, TAZ and FOXO3 proteins have been examined by immunohistochemistry staining.

Also, in the revised figures, we followed the previous design, and the tables containing the detailed information are made, which would provide the evidence for research studying the deeper mechanism of primordial follicle formation. For instance, the results showed the “Ferroptosis” maybe another apoptosis way for oocyte loss (supplemental Table 10).

19.	Fig S6: the violin plots suggest cross-contaminations between the granulosa populations and the oocyte populations. For instance, Kit is detected in some granulosa cells and Kitl is detected in some oocytes. Same with Gdf9, detected in some granulosa. These data suggest a problem with the single cell data, probably at the cell dissociation step. This likely impacted the clustering and data analyses.

**Answer:** Thanks for your comments. In the revised manuscript, to avoid the cross-contaminations issue, the strict quality control and careful check are both applied. On the one hand, the cells with excessive nFeature_RNA are believed as potential multiple lets and they are discarded. In another, the cells high expressed marker gene of other cell types are discarded. At the same time, the distribution of nFeature_RNA in one cell type is also checked to avoid cross-contaminations. However, one condition that cells wrapping the dissociative RNA of single cell solution due to cell disruption during the process of tissue digestion could potentially trigger a slight certain extent contamination, which still be an unsolved issue. Maybe that’s the reason why a little bit of Kitl can be detected in some oocytes in our study after applying the available method above.

20.	This manuscript lacks mention/definition of atretic germ cells or somatic cells during the time course. How were these cell types taken into consideration into the analyses? Were the atretic cells omitted entirely? Please address in the discussion.

**Answer:** Thanks for your comments. In our study, we found that the number of germ cell largely decrease, which likely attribute to atretic germ cells. In the workflow of single cell sequencing of 10 x Genomics, it requires the survival cells for capture and library construction. Thus, we speculate the apoptotic cells can not undergo a series of PCR and sequencing like normal cells. So, the atretic cells may be omitted entirely as low-quality cells in the pre-process. We also will add the information in the discussion.

*“Given the fact of germ cell loss, apoptosis has been the most commonly identified process; however, the present scRNA-seq platform does not excel in the determination of apoptotic cells, and dying cells are regarded as low-quality and therefore need to be discarded; another known is mechanism of cell autophagy, which was enriched (Fig 7A), it is noteworthy that ferroptosis, one form of cell death, was revealed in the current study (S10 Table), and its roles in oocyte loss deserves to be investigated further..”*

**Editorial comments:**

Line 50: The ovarian reserve is not established yet at birth, but rather about a week after birth. At birth, formation of all primordial follicles is not complete and some oocytes are being lost through attrition.

**Answer:** Thanks for your comments. The problem has been revised. The sentence “At birth” here is replaced by “After establishment within one week…”

Line 141: you need to explain what canonical correlation analysis is so that the reader understands the meaning of these results. There is not sufficient information on Figures S1D and S1E to understand what they represent.

**Answer:** Thanks for your comments. As reported, CCA aims to find linear combinations of features across data sets that are maximally correlated, identifying shared correlation structures across data sets. In the previous report, we perform the CCA analysis for the integration of three data sets in our study, which is embedded in Seurat V2 pipeline. Figures S1D and S1E showed the distribution of cells in each sample under CC1 versus CC2 condition. In the following steps, we align the CCA subspaces to integrate the three samples, which returns a new dimensional reduction called “cca.aligned” that can be used for downstream analyses such as clustering.

In fact, the new strage has been applied for an integrated analysis, that is “anchors”, which was implemented in Seurat V3. The unified strategy is presented for reference assembly and transfer learning for transcriptomic, and spatially resolved single-cell data. Through the identification of cell pairwise correspondences between single cells across datasets, termed ‘‘anchors’’[1], it can transform datasets into a shared space and enables the construction of harmonized atlases at the tissue or organismal scale, as well as effective transfer of discrete or continuous data from a reference onto a query dataset.

**Reference:**

[1] Stuart T, Butler A, Hoffman P, et al. Comprehensive integration of single-cell data[J]. Cell, 2019, 177(7): 1888-1902. e21.

Line 161: the terms “late-follicular” / “Late follicle” are inappropriate and could be confusing since the latest stage analyzed here, P3, still represents very early stages of folliculogenesis. The authors should use a different terminology for the stages of oocyte development they describe in figure 3/4. Maybe “Pre follicle / early follicle formation / late follicle formation”.

**Answer:** Thanks for your comments. As suggested, we have revised the description.

Fig.4E, 5B-E-F, 6A, 7D-E-F-H: the axis or legend of the graphs are not visible/too small.

**Answer:** Thanks for your comments. Here, we have adjusted the text size. Also, the check of whole manuscript has been made.

Line 195: “Conversely, ClueGO functional networks of genes in cluster 3 and 4 under regulated in germ cell cysts…” Cluster 3 represents genes highly expressed in germ cell cysts as mentioned line 183. Clusters 3 and 4 have completely different profiles, merging them for gene network analysis does not make sense.

**Answer:** Thanks for your comments. In re-submitted figure, the functional network of gene cluster has been revised according to your advice. As shown in Figure S2F, the genes both up-regulated of germ cells within cyst (gene sets 1 and 2) are merged to perform the KEGG enrichment analysis, and Figure S2G are gene both highly expressed in germ cells within the follicle (gene sets 4 and 5).

Line 246: typo Cdkn1c.

**Answer:** Thanks for your correction. We have corrected the typo error.

The single branch point in the pseudo-time analysis of the granulosa cells was not well-defined. Please define the potential biological significance of the single branch point.

**Answer:** Your comments are very important. In fact, we define the single branch of granulosa cells with both considerations of expressed gene signature and time point of cells.

*“…, Cell pseudotime trajectories revealed three granulosa cell States and one branch point (Figs 6B and 6C). Following the considerations reported in the results, State 1 was attributed to early pre-granulosa cells, it branched at State 2 of pre-granulosa cells expressing Lgr5 and Gng13, and at State 3 of late stage pre-granulosa cells expressing Foxl2 and Hsd17b1 that are required for steroidogenesis. As recently reported [47], there are two granulosa cell differentiation pathways supporting follicle formation: State 3 cells with high expression of Foxl2, Aard and Akr1c14 are suggested to be bipotential-derived pre-granulosa cells supporting follicle in the ovarian medulla; meanwhile, the epithelial pre-granulosa cells are for oocytes destined for the ovarian cortex, as evidenced by the expression of Lgr5, Gng13 and Krt19 at State 2 (Fig 6D).…”*

Numerous bioinformatic approaches were taken to analyze the data, but many graphs are not explained nor contribute to significant discussion points such as Figure 4E, 5B, 6C, and 6D. Replace these figures to the supplementary or discuss the significance of these graphs’ data.

**Answer:** Thanks for your suggestions. We have added the interpretation for the graphs in the newly revised manuscript.

As Figure 4E and 5B showed, they displayed the dynamic changes of gene expression in different gene sets. In the revised manuscript, in like manner, we showd the dynamic expression of each gene set regard to the germ cell fate in Figure 4E.

*“In such pseudotime trajectories, three branches implied three differentiated fates of germ cells, and five gene clusters with distinct patterns were identified, which likely involved in the commitment of cyst germ cells into follicular oocytes (Supplemental Table 3); clusters 1, 2, 3, 4 and 5 including 722, 1819, 359, 1077 and 1904 genes, respectively (Figures 4D and 4E)…The expression kinetics of each gene set are shown in Figure 4E…”*

In Figure 6C, it involves all the regulons (TFs and their targeted genes) that matched with cell status and their transcriptome signature. Meanwhile, the regulon activity is be binarized with “0” to “1”, “0” present “off” of TFs, and “1” refer to “on”. Thus, the Figure 6C demonstrates the whole landscape of TFs activity involved in germ cell development during the process of primordial follicle formation in data set perspective. Among, four TFs, including CUX1, CREB1, GTF2F1 and NELFE, are always “on” throughout the detected stage, Figure 6D showed their expression patterns. In the new figure, we place emphasis on TFs related with follicle assembly, the expressions of the TFs have been move into supplementary as you suggest.

*“As shown, a series of TFs displayed a more dynamic pattern. For example, Nr3c1, Brca1, Kdm5a and Kdm5b were active mainly at the germ cell nest stage (Fig 5B and S3C Fig), and Klf7 and Gabpa seemed to be mostly activated at the nest stage, but gradually turned off as follicular assembly occurred (Fig 5C). Similarly, Smarcb1, Hes1, Sox15 and Stat3 were mainly associated with States 2 and 3, corresponding to the period of primordial follicle assembly (Fig 5D and S3D Fig). Furthermore, a portion of TFs appeared to serve as a transient switch, such as Foxo1 had a limited role in germ cells within nests and Foxo3 functioned during a period of the follicular stage (Fig 5E). Moreover, several TFs, such as Jun and Klf2, were significantly represented during all oocyte stages, which supports a role for these TFs as likely candidates for sustaining a germ cell specific transcription program throughout the investigated period (S3E Fig).”*

The final figure, Figure 9, is not clear. The legend on the bottom indicating expression levels between high and low in orange and green are not clearly represented in the figure. The study’s time course should be included (E16.5, P0, P3).

**Answer:** Thanks for your comments. The modification of the legend has been done accordingly. In fact, the legend of expression levels between high and low in orange relects the expression dynamics of marker genes in germ cell, and green are pre-granulosa cell. Also, we add the time course in the gragh.

*“The upper panel indicates the genetic programs and key biological events of germ cells and pre-granulosa cells during germline nest breakdown and follicle assembly, the developmental time courses are indicated above, the orange box contains the marker genes and their expression dynamics in germ cell; the green ones comprise the marker genes and their expression dynamics in pre-granulosa cell. The dark orange dashed line separates ovarian cortex (above) from the medulla, labeled with two sources of pre-granulosa cells, that is bipotential pre-granulosa (BPG) cells for 1st wave in ovarian medulla and epithelial pre-granulosa (EGP) cells in ovarian cortex for the 2nd wave follicle formation, respectively. In the below panel (upper part), the activation of five main interactive pathways between germ cells and granulosa cells during these processes is represented. In the lower part, the stage-specific transcription factors in oocytes from nest to follicle stage are reported.…”*

Within the manuscript, vague terminology such as “they” and “these” are used and should not be utilized in scientific writing. Remove and replace the use of these words. Lines 126, 284, and 361.

**Answer:** Thanks for your comments. The error has been revised, and we also check the whole the manuscript to improve the writing. To make is more scientific, the professional scholar has been invited to revise and polish the manuscript.

A conclusion paragraph is lacking and must be included in the discussion. Specifically address the overall impact of the research, how these data compare to other scRNAseq studies (such as Nef), the overall strengths and weaknesses of this paper, and what else needs to be done in order to address the molecular control underlying primordial follicle assembly.

**Answer:** Thanks for your comments. We have added the summary of conclusion paragraph in the discussion.

*“… however, the present scRNA-seq platform does not excel in the determination of apoptotic cells, and dying cells are regarded as low-quality and therefore need to be discarded…”*

*“In summary, the present study highlighted the transcriptional dynamics taking place in oocytes and granulosa cells during follicular formation by scRNA-seq. Specifically, the development stage-specific marker genes of germ cells and granulosa cells are identified. Furthermore, the transcriptional factor regulatory networks of germ cells and novel pathways involved in primordial follicle formation have been given preliminarily confirmation. Thus, the current study provides novel insights into follicle formation at the perinatal stage and offers a reliable resource for study of the molecular regulatory mechanism of follicular development in the mouse.”*

*“Furthermore, despite of the fact that the single cell transcriptome technique is greatly expanding our insights into biological systems, especially regarding cellular heterogeneity, there are some unsolved issues that still need addressing within the field. Firstly, it’s the current lack of standardization for date analysis [75]. The problem is triggered by the growing number of analysis methods, exploding dataset sizes, and technical aspects (such as a variety of programming languages for data analysis tools), all of which prevent computational biologists from developing a gold standard practices. Another is regard to the incorporate of biological replicates [76, 77]. Although the biological replicates are suggested to eliminate batch effects, it seems the one replicate is still more popular in the single cell dataset of recent publications [47, 61]. In the present study, the application of one replicate may be considered as a potential limitation, however, the canonical knowledge in the field of primordial follicle formation, as well as the experimental verification, make us confident for our results. Moreover, the cell proportions studied here, at least partially, do not reflect the true cell type compositions in ovarian tissue (such as germ cells), this may be attributed to the methods of tissue handling and dissociation for single cell sample preparation, which is also present in another publication of single cell transcriptome investigating the female ovary [78].”*

In figures 2D and 3E, please including a legend defining the size of the dots in the dot plot.

**Answer:** Thanks for your comments. Yes, the lacking legend has been added to make reader to understand the means of graph more easily, such as in revised Figure 2D. And also see its Legend:*“Dot size represent the cell percent expressed in cluster; dot color marker the average expression level of gene.”*

Line 226, it is not clear what the authors mean “…despite their expression being limited to specific regions.” Please elaborate as Figure 6C appears to show that the four transcription factors are throughout the heatmap.

**Answer:** Thanks for your comments. Followed amount adjust of figures, we largely revised the manuscript. As stated here, the four transcription factors are throughout the heatmap (Figure 6C), in fact, the heatmap showed the regulon activity, not only TFs, but also includes their targeted genes, in Figure 6D, it showed the TFs expressions seem to be limited to specific regions.

In the revised Figure, the SCENIC results are showed in Figure 5. Similarly, regulon activity was binarized and matched with germ cell State and developmental stages (left panel in Fig 5A). Also, several TFs, such as Jun and Klf2, were significantly represented during all oocyte stages, which supports a role for these TFs as likely candidates for sustaining a germ cell specific transcription program throughout the investigated period (S3E Fig).

Line 383, please elaborate “…gene expression changes are not always essential.” as this manuscript focuses on RNA sequencing data.

**Answer:** Thanks for your comments. The saying here is to express that the result of manuscript focuses on RNA sequencing data, which only provide the gene expression changes at mRNA level, the further analysis is required to detect the function of key gene involving in follicle formation. Thus, it suggested “…gene expression changes are not always essential”. Thanks all the same.

Lines 427 – 432 is an unnecessarily long sentence that needs to be broken up.

**Answer:** Thanks for your comments. We have revised the sentence.

Figure S2C, the scale values are not clear and overlap each other.

**Answer:** Thanks for your comments. The scale values are adjusted to proper size.

Figure 1A, provide scale bar for zoomed in IF images on the far right.

**Answer:** Thanks for your correction. We have added the scale bar information.

Figure S5C, the scale value is not defined and appears to be missing a value.

**Answer:** Thanks for your comment, we have checked and revised the scale value.

Line 841, do the authors mean “Venn diagram” and not “Vlnplots”?

**Answer:** Thanks for your correction. It should be “Venn diagram”.

The legend in figures 4E and 5B are illegible. Increase the size of the figure legend.

**Answer:** Thanks for your comment. The figure legends have been revised with larger text size
