# Supplementary material for: Single-cell transcriptome landscape of ovarian cells during primordial follicle assembly in mice
Source: PLoS Biol. 2020 Dec 22;18(12):e3001025. doi: 10.1371/journal.pbio.3001025 (PMC7787681; doi:10.1371/journal.pbio.3001025)
Supplement: Attachment — Submitted filename: Reply to Reviewers_R3.docx [file pbio.3001025.s022.docx]

**Publication editor:**

1. Many thanks for citing your Github deposition in the legends for your main Figure. Please could you also add the same statement (“Panels in this Figure can be reproduced using data in GSE134339, using scripts in https://g 639 ithub.com/WangLab401/2020scRNA_murine_ovaries”) to the relevant supplementary Figure legends?

**Reply:** Thank you very much, as suggested, we have added the similar text in the relevant supplementary figure legends of our manuscript.

2. Sorry for the lack of clarity about the two files S2_Data and S3_Data. I can see why you were confused by my request, but I am also confused. In each of these two files, there are three tabs, named "Fig 1B," Fig 1F" and "Fig 1l." We do not understand what these tab names mean, and how they relate to the Figures in your paper. Please could you clarify?

**Reply:** As for the issue, we finally realize the reason of the problem. A sincere apologize from us, we have revised the tabs and resubmitted these two tables. Thanks a lot

==============================================

We would appreciate your prompt cooperation with our requests at your earliest convenience. If you have any questions or require clarification, please do not hesitate to get in touch.

**Reply:** We very appreciate about your letter. An apologize for missing attention about the request from the previous e-mails. Here, we concentrate on the manuscript requirements and the point by point reply has been listed below. Thanks for your work regard to our manuscript again.

MANUSCRIPT REQUIREMENTS

Please update your manuscript with the following changes:

1. We do not publish any copyright or trademark symbols that usually accompany proprietary names, eg (R), (C), or TM (e.g. next to drug or reagent names). Therefore please remove all instances of trademark/copyright symbols throughout the text, including '®' (Fluor ®) on page 27 of your manuscript file.

**Reply:** Thank you, we have removed the special symbols from our manuscript;

2. Please indicate the corresponding author on the title page with an asterisk (*) and include the corresponding author's email address. Only an asterisk and the email address itself are required.

Please format as follows, omitting any other name, address or contact information:

* wshen@qau.edu.cn; shenwei427@163.com

**Reply:** Yes, the format of corresponding author information has been revised as requested;

3. Please ensure that all authors have at least one affiliation listed on the title page and that all listed affiliations are attributed to at least one author. Affiliations should be listed in ascending numerical order in the affiliation list and should match what is provided in the Authors Information in the submission form.

Affiliations should include an institution, a city, and a country (no postcode). US affiliations need the state as well, written out in full (with no abbreviations).

**Reply:** It’s okay. The information of Affiliations has been checked, as suggested, the postcode is omitted and the abbreviations of state name are replaced with full name;

4. Please ensure that your Ethics Statement is available in the Methods section of your manuscript in its entirety, under the subheading “Ethics Statement”.

**Reply:** The remind in nice. We have added the subheading of “Ethics Statement” and adjusted the relevant content;

5. We have noted that you have the following present in your manuscript: List of keywords/ Financial Disclosure/ Competing Interests statement/ Author Contributions/ Data Availability Statement

We will remove these section from your manuscript file as this information is automatically pulled from the submission form. Please make sure that the information in the submission form is correct.

**Reply:** Thank you, the required sections are checked in our manuscript and they are right;

6. Please make sure all references are cited in ascending numerical order in the text.

**Reply:** All the references are checked in our manuscript and they are right, thanks again;

7. Please upload your Supporting Information items individually, rather than in a single file, as you refer to them individually in the text. If you would like to retain the single file please rename internal items (e.g. tables) as A, B, C etc. Please also provide a legend for each under the heading Supporting Information. Supporting Information files should be less than 10 MB each. Please edit any citation of internal items within your Supporting Information files to using letters rather than numbers, e.g. “Table A in S1 Text”. Please ensure these changes are made throughout the manuscript and within the Supporting Information files themselves.

8. Please ensure that all Supporting Information files use the following format verbatim throughout the manuscript (including legends and all in-text citations): S1 Fig, S1 Table, S1 Text, S1 Data, etc. Each file type should start at 1 and increase numerically. Please note, however, that the file names themselves must use an underscore rather than a space, i.e. S1_Fig.tif, S1_Table.xlsx, S1_Text.doc, S1_Data.xlsx, S1_PRISMA_Checklist.docx, S1_Zip.zip, etc.

9. Please provide a legend for your Supporting Information zip file at the end of your manuscript, along with all of your other Supporting Information file legends. Refer to the zip file as ‘S1 Zip’ in the manuscript text.

**Reply:** Q7-9. Many thanks. The Supporting Information files have been uploaded individually, and the format citations in the manuscript have followed the suggestion, as well as the legends and file names;

10. Many thanks for supplying the Github URL for your R code, and for depositing your raw data. Can you confirm that this would enable a reader to reproduce the Figures in your paper? If so, please include the following (or similar) text in each relevant main and supplementary Figure legend: “Panels in this Figure can be reproduced using data in GSE134339, using scripts in https://g 639 ithub.com/WangLab401/2020scRNA_murine_ovaries”

**Reply:** We very appreciate it. We have made the files available to reproduce the Figures in our manuscript. The similar text has been added in each relevant main Figure legends;

11. Thank you for providing S2_Data and S3_Data; however, these do not seem to have a simple relationship to Figs 3D and S1B, as claimed (specifically, the “Fig 1l” tab of each file corresponds to the Figure panels, but the nature of the “Fig 1B” and “Fig 1F” tabs is unclear. Please could you clarify?

**Reply:** Thank you very much. The S2 Data and S3 Data have been revised and resubmitted, in our previous files, the values of each independent repeat were listed, in the revision, they have been replaced with mean value, and they are more direct with the Figure; Another, we have a little confuse about your next query, whether the Fig 1B tab refers to the legend, and we also have no idea about the “Fig 1l” and “Fig 1F”, that not included in our Figures and Manuscript. No matter how, we have revised the Fig 1B Legend.

*“UMAP plot of ovarian cells colored by the selected developmental time points (that is Stage). E16.5 for mediumorchid, PD0 for lightblue and PD3 for darkcyan.”*

FIGURE REQUIREMENTS

Please complete the following instructions regarding your figure files:

1. Please confirm that you either created the following figure panels, or that you will replace them with an open source alternative: illustrations in Fig 1

Here are a few open source image websites you may wish to use:

- https://commons.wikimedia.org

- http://www.publicdomainpictures.net/

- https://openclipart.org/

**Reply:** Here, we confirm that the illustrations in Fig 1 is drew by ourself. The component is created and slightly adjusted by imitating the relevant image on the internet. Thank you all the same.

We have made a revision in our manuscript following the above request, and the format has been improved well, we hope it will make you satisfied. Thanks a lot.

**Editor：**

I have now obtained advice from two of the original reviewers and have discussed their comments with the Academic Editor. Based on the reviews, we will probably accept this manuscript for publication, assuming that you will modify the manuscript to address the remaining points raised by the reviewers. Please also make sure to address the data and other policy-related requests noted at the end of this email.

IMPORTANT:

a) Please attend to my Data Policy and Ethics Policy requests further down the email.

b) Please could you add one or two sentences to your Abstract, stating what novel biological insights are provided by this study (or might be provided by further studies that use this data)?

c) Please address the remaining concerns raised by the two reviewers.

**Reply:** Thanks very much for your work about our study. Much attention has been paid to meticulously handle the concerns raised by the two reviewers, also the abstract has been well optimized. Additionally, the data and other policy requests are well conducted. We appreciate all the efforts you invested in our study, thanks again.

*“… and the interactions of germ cells and granulosa cells basing on known and novel pathway were presented. … Above all, this study provides the whole transcriptome landscape of ovarian cells and unearths new insights during primordial follicle assembly in mice.”*

We expect to receive your revised manuscript within two weeks. Your revisions should address the specific points made by each reviewer. In addition to the remaining revisions and before we will be able to formally accept your manuscript and consider it "in press", we also need to ensure that your article conforms to our guidelines. A member of our team will be in touch shortly with a set of requests. As we can't proceed until these requirements are met, your swift response will help prevent delays to publication.

**Reply:** Thank you very much. The revisions have been completed in time. Further, our article has been checked and modified according to the journal guidelines. We hope the newly revision make you satisfied.

To submit your revision, please go to https://www.editorialmanager.com/pbiology/ and log in as an Author. Click the link labelled 'Submissions Needing Revision' to find your submission record. Your revised submission must include the following:

- a cover letter that should detail your responses to any editorial requests, if applicable

- a Response to Reviewers file that provides a detailed response to the reviewers' comments (if applicable)

- a track-changes file indicating any changes that you have made to the manuscript.

**Reply:** Thank you. We would submit the revision following your request.

*Copyediting*

Upon acceptance of your article, your final files will be copyedited and typeset into the final PDF. While you will have an opportunity to review these files as proofs, PLOS will only permit corrections to spelling or significant scientific errors. Therefore, please take this final revision time to assess and make any remaining major changes to your manuscript.

NOTE: If Supporting Information files are included with your article, note that these are not copyedited and will be published as they are submitted. Please ensure that these files are legible and of high quality (at least 300 dpi) in an easily accessible file format. For this reason, please be aware that any references listed in an SI file will not be indexed. For more information, see our Supporting Information guidelines:

https://journals.plos.org/plosbiology/s/supporting-information

**Reply:** The remind is very kind. The best efforts have been invested to check our manuscript in spelling and scientific writing to make it perfect. Also, Supporting Information files with high quality has been submitted. Thanks a lot.

*Published Peer Review History*

Please note that you may have the opportunity to make the peer review history publicly available. The record will include editor decision letters (with reviews) and your responses to reviewer comments. If eligible, we will contact you to opt in or out. Please see here for more details:

https://blogs.plos.org/plos/2019/05/plos-journals-now-open-for-published-peer-review/

**Reply:** The suggestion is nice. We are quite willing to make review history publicly available, which may be conductive to the understanding for readers.

*Early Version*

Please note that an uncorrected proof of your manuscript will be published online ahead of the final version, unless you opted out when submitting your manuscript. If, for any reason, you do not want an earlier version of your manuscript published online, uncheck the box. Should you, your institution's press office or the journal office choose to press release your paper, you will automatically be opted out of early publication. We ask that you notify us as soon as possible if you or your institution is planning to press release the article.

**Reply:** Thank you. We agree with the release of our early manuscript.

*Protocols deposition*

To enhance the reproducibility of your results, we recommend that if applicable you deposit your laboratory protocols in protocols.io, where a protocol can be assigned its own identifier (DOI) such that it can be cited independently in the future. For instructions see: https://journals.plos.org/plosbiology/s/submission-guidelines#loc-materials-and-methods

**Reply:** Thank you. We would like to share the information. However, as for our study, the major of figures are derived from data analysis of single cell sequencing. Here, we refer to the recent publications of the same type on PLOS BIOLOGY (such as https://doi.org/10.1371/journal.pbio.3000538), and the R code is provided publicly. Here, we also have made the sequencing raw data and source code using in the study available in public repository (Dataset on NCBI GEO: GSE134339; R code at GitHub https://github.com/WangLab401/2020scRNA_murine_ovaries.). The experiment in laboratory only involves conventional experimental techniques, including western blot and immunohistochemistry, which are both well presented in Method of manuscript. Hope you are satisfied with this scheme. Thank you all the same.

ETHICS STATEMENT:

Your ethics statement says "“Animals were housed according to the national guideline and Ethical Committee of Qingdao Agricultural University.” My understanding is that all that you did was to mate the mice and then sacrifice them, which would need no further approvals, but please could you specify how the mice were sacrificed?

**Reply:** In our experiment, the main sample are murine ovaries. At embryonic 16.5 and postnatal day 0 and 3, the pregnancy mice and pups were killed in the manner of necks broken to isolate ovarian samples, respectively. According to the relevant management regulations of experimental animals, all the experimental operation on animals should been approved by Institution of Animal Care and Ethical Committee. Here, we made a supplement in the manuscript.

“*Animals were housed according to the national guideline, the mice were sacrificed by cervical dislocation and other experimental operations on mice have been approved by Animal Care and Ethical Committee of Qingdao Agricultural University.*”

DATA POLICY:

You may be aware of the PLOS Data Policy, which requires that all data be made available without restriction: http://journals.plos.org/plosbiology/s/data-availability. For more information, please also see this editorial: http://dx.doi.org/10.1371/journal.pbio.1001797

Note that we do not require all raw data. Rather, we ask that all individual quantitative observations that underlie the data summarized in the figures and results of your paper be made available in one of the following forms:

1) Supplementary files (e.g., excel). Please ensure that all data files are uploaded as 'Supporting Information' and are invariably referred to (in the manuscript, figure legends, and the Description field when uploading your files) using the following format verbatim: S1 Data, S2 Data, etc. Multiple panels of a single or even several figures can be included as multiple sheets in one excel file that is saved using exactly the following convention: S1_Data.xlsx (using an underscore).

**Reply:** Thank you, we would prepare the supplementary files and submit the data materials according to the data policy.

2) Deposition in a publicly available repository. Please also provide the accession code or a reviewer link so that we may view your data before publication.

**Reply:** Thank you, we have submitted the supplementary raw data files, as well as the raw sequencing data and source code during data analysis, also all of them are publicly available. Thank you very much.

*“The accession number of ovarian single-cell RNA sequencing raw data reported in this paper is NCBI GEO: GSE134339. The R code using for data processing is publicly available at GitHub at* [*https://github.com/WangLab401/2020scRNA_murine_ovaries*](https://github.com/WangLab401/2020scRNA_murine_ovaries)*.”*

Regardless of the method selected, please ensure that you provide the individual numerical values that underlie the summary data displayed in the following figure panels as they are essential for readers to assess your analysis and to reproduce it: Figs 1BCDE, 2ABCD, 3D, 4ABCDEG, 5ABCDE, 6ABCDEFG, 7ABCDEFGH, S1BDE, S2ABCDEFG, S3ABCDE, S4ABCD, S5ABCD, S6BCDEFG, S7ABCDE. NOTE: the numerical data provided should include all replicates AND the way in which the plotted mean and errors were derived (it should not present only the mean/average values).

Please also ensure that figure legends in your manuscript include information on where the underlying data can be found, and ensure your supplemental data file/s has a legend.

Please ensure that your Data Statement in the submission system accurately describes where your data can be found.

**Reply:** Thank you, we have carefully checked the figures and their legends in the manuscript, and the detail information that required have been modified.

BLOT AND GEL REPORTING REQUIREMENTS:

For manuscripts submitted on or after 1st July 2019, we require the original, uncropped and minimally adjusted images supporting all blot and gel results reported in an article's figures or Supporting Information files. We will require these files before a manuscript can be accepted so please prepare and upload them now. Please carefully read our guidelines for how to prepare and upload this data: https://journals.plos.org/plosbiology/s/figures#loc-blot-and-gel-reporting-requirements

**Reply:** Thank you, according to the requirement, we have uploaded the original figures in the attached files.

**REVIEWERS' COMMENTS:**

**Reviewer #2:**

The authors have addressed my comments satisfactorily and I believe the revised manuscript is much strengthened. I just have one minor request which is that they authors integrate the complete rationale for the three ages used for collecting cells in the actual manuscript text. In the reviewer response, the authors provided an important explanation about the relevant biology which is lacking in the actual manuscript text.

**Reply:** Thanks a lot. As you suggest, we have replenished the explanation regard to ovarian ages in Method text (as bellow). Thanks for your advice again.

“*The selected three time points for sequencing represent the key stage of a series of cellular events involving primordial follicle formation, i.e. E16.5 time point represents the germ cells preparation (see prerequisites cellular events for the establishment of PFs [15]), and PD0 is the initial time point of nest breakdown, when it comes to PD3, more primordial follicles have formed.*”

**Reference:**

[15] Wang C, Zhou B, Xia G. Mechanisms controlling germline cyst breakdown and primordial follicle formation[J]. Cellular and Molecular Life Sciences, 2017, 74(14): 2547-2566.

**Reviewer #3:**

In the revised manuscript, the authors took into account our comments and completely reanalyzed their single-cell datasets, resulting in a significantly changed manuscript.

A major concern about the previous version was that the scRNA-seq data clustering of the cell populations was not reflecting the biology of the ovary. Particularly, some clusters appeared to be composed of a mixture of different known cell populations, which skewed all the downstream analyses of these clusters. It resulted in some conclusions that were not supported by what is already known in the field. In this new version of the manuscript, the authors used more stringent settings/new methods to reanalyze their scRNA-seq data. It greatly improved the identification of the cell populations and downstream analyzes of each population. Consequently, all the figures of the manuscript are completely changed. The authors also took in account the reviewers comment of focusing on novelty rather than what is already known. A validation for new markers for identified sub-population of cells is now provided (new Figure 3).

The new combined analysis of germ cell and granulosa for identification of cross-talks of ligand/receptor of common signaling pathways in Fig. S5 is interesting, although it could have been used to characterize new and less known pathways in addition to the already well characterized pathways presented here. Some validation of these findings through immuno-fluorescence or ISH would enhance the confidence of the scRNA-seq findings.

The authors also provide more detailed explanations and definitions throughout the manuscript so that non-expert readers can better understand some sc-RNA-seq jargon used. Overall, the authors have greatly improved the manuscript, for the analysis of the data, their interpretation and explanations of the results.

**Reply:** Thank you very much. Indeed, to make our study more scientific, the datasets are completely reanalyzed and a large amount of modifications has been made in the manuscript. The work has been improved immensely due to your professional review comments. We also benefit a lot. Thanks again.

My specific comments are:

1. Line 29: "the complete dynamic genetic programs of germ and granulosa cells from E16.5 to PD3 were reported for the first time, to our knowledge": This is an overstatement as a recent publication by Niu and Spradling have provided a time course of sc-RNA-seq in ovaries at E11.5, E12.5, E14.5, E16.5, E18.5 P1, P5 and also included specific analyzes of both germ cells and granulosa cells (Reference 47 in the manuscript). Since the current manuscript and this published paper both focus on very similar subjects, it would be relevant to have a few sentences in the discussion that specifically compares findings between the 2 manuscripts.

**Reply:** The comment is great. Here, we have changed the saying in Abstract. In addition, we also replenished the compares between our work with that of Niu and Spradling.

*“Since continuously, the origin of granulosa cell is always elusive, which was hypothesized to be derived from mesonephros or ovarian surface epithelial cells [66]. As recently reported [47], there are two granulosa cell differentiation pathways supporting follicle formation, which are marked by Foxl2 and Lgr5 expressions, and they show different transcriptional patterns and are involved in two waves of follicle formation. In our results, the two distinct cell populations above were indeed confirmed, analogously, State 3 cells with high expression of Foxl2, Aard and Akr1c14 are suggested to be bipotential-derived pre-granulosa cells supporting follicle in the ovarian medulla; meanwhile, the epithelial pre-granulosa cells are for oocytes destined for the ovarian cortex, as evidenced by the expression of Lgr5, Gng13 and Krt19 at State 2 (Fig 6D). Thus, the consistent results in two study further evidenced the notion that two kinds of pre-granulosa cells supporting follicle formation respectively at ovarian medulla and cortex in mice.”*

2. Line 135: "Furthermore, the cell cycle phase score result showed the germ cells heterogeneity was not interfered by cell cycle genes expression (S2A Fig)": At E16.5-P3 the germ cells have all initiated meiosis. Why the analysis based on cell cycle when germ cell are in meiosis?

**Reply:** Thank you for the comment. Here, the cell cycle scores analysis was performed to test whether the germ cells are dependent on the cell cycle genes expression or not. During the investigated periods, it’s reported that most germ cells enter the diplotene of meiotic prophase I postnatally [1], which indicate that germ cells may be associated with cell cycle genes, at least partly, especially for cells at E16.5 and PD0, although the fact is not actually like that through our analysis (S2A Fig). Meanwhile, with secondary consideration, we finally decide to remove S2A Fig for its less significance.

Reference:

[1] Burks D M, McCoy M R, Dutta S, et al. Molecular analysis of the effects of steroid hormones on mouse meiotic prophase I progression[J]. Reproductive Biology and Endocrinology, 2019, 17(1): 1-10.

3. I find the pseudotime analyses misleading, particularly on the interpretation of what the sub-populations of both germ cells and granulosa cell actually are. For germ cells, the authors stated in Line 160 "In such pseudotime trajectories, three branches implied three differentiated fates of germ cells". This definition does not apply to these sub-populations of germ cells. There is continuous differentiation into these sub-populations, from State 1 to State 3 (although it is unclear what State 2 actually represents). State 1 and State 3 are not different "fates of differentiation", but rather different "stages of differentiation" within the same population. State 1 is defined as "Nest" germ cells and state 3 is defined as "Follicle" germ cells. What does State 2 represent? Is it an intermediary state between state 1 and 3, or is it possible it could represent some pre-atretic germ cells? Gene set 3 seems to be specific of this State 2 and includes GO terms related to "cellular response to DNA damage" (Fig. 4D-E).

**Reply:** The comments are very good. As suggested, the modification has been made in our manuscript.

Firstly, in pseudotime analysis of germ cells, the saying of “fates of differentiation” has been changed as “stages of differentiation”.

Secondary, regard to cells at State 2, we prefer to define them as an intermediary state between state 1 ("Nest" germ cells) and 3 ("Follicle" germ cells), which marked with high expression of key genes essential for follicle assembly, such as Lhx8 and Sohlh1 in get sets 1 and 2. At the same time, the cells at State 2 consisted of cells in clusters 3 and 4 (Figure 4B and 2D), which are viewed as early stage of follicle formation.

In another aspect, it remains to be seen whether the State 2 cells were apoptotic. Here, the gene set 3 have enriched GO term of “cellular response to DNA damage”, however, it locates at a lower enrichment level with “log10(P) = -3.32”, to be note, part of these genes also highly expressed in “Nest” germ cells. As we known, homologous chromosomes recombination occurs during meiotic prophase Ⅰ at the investigated periods. In the process, the event of a programmed induction of DNA double-strand breaks happen, and the relevant genes are likely enriched at GO term of DNA damage, correspond to that, gene set 5 also enriched GO term of “DNA repair”, which are highly expressed in “Nest” germ cells, these results imply DNA damage maybe refer to meiotic damage. Thus, we can’t judge these cells as “possible apoptosis” with no other more evidence. Thank you again.

4. Following comment 3 for granulosa cells, the State 1, which were defined as "Early PGs", are actually BPGs, but at a less differentiated state than State 3 labelled "BPGs". Both represent wave 1 of granulosa cells. Maybe it would be more appropriate to name State 1 "Early BPGs" and State 3 "Differentiating BPGs". State 2 "EPGs" is defined line 225 as "differentiated pre-granulosa cells". On the contrary, these cells represent wave 2 of granulosa cells that are at an even earlier stage of differentiation: these cells are currently recruited from the epithelium and do not express Foxl2 yet in contrary to States1-3, but they eventually will to form the cortical follicles.

**Reply:** Thanks for your comment. In the revised manuscript, we have renamed State 1 cells with “Early BPGs”, and State 3 for “Differentiating BPGs”. State 2 was renamed as “Differentiating EPGs”.

5. Fig. 5A-E: what do the numbers in parentheses next to the regulons mean? Ex: "Foxo3 (16g)". It is not defined in the legend.

**Reply:** The comment is timely and great. The numbers in parentheses next to the regulons is the number of gene in regulons. We have added the information in the legend of Fig 5A.

*“The numbers in parenthesis next to the regulon names indicate the number of genes enriched in regulons.”*

6. Line 214: "For example, Wnt4 expression was most activated at E16.5, which may imply the differentiation of ovarian pre-granulosa cell and preparation the secondary follicle formation [19,40]." I don't understand this sentence. What is the link to secondary follicle formation?

**Reply:** Thanks for your comment. We have revised the sentence of “secondary follicle formation” with “second wave of follicle formation”. Accordingly, REPO1/WNT4 signal is vital to the differentiation of ovarian pre-granulosa cell. It has been reported that the BPG cells recruited around E11.5 supported the first wave of follicle formation, which is dependent on REPO1/WNT4 signal (ref 19). From E12.5 to E16.5, it also been proposed REPO1/WNT4 signal is required to establish the Lgr5+ population (ref 40), that support the second wave of follicle formation, thus, the high expression of Wnt4 here is supposed to get ready for the second wave of follicle formation.

7. Fig. S6C. I don't understand the point of the combined clustering of granulosa and germ cells when there is already the transcriptomic data for each separated population.

**Reply:** Thanks for your comment. In fact, we integrated both the granulosa and germ cells to display the difference of pathway members in two cell types in a comparative way, thus, the Fig S6C has been delete because its useless. As shown in Fig 7C, the dot plots could well present the comparative expression of FoxO factors on germ cells and granulosa cell, for example, the expressions of *Foxo1* and *Foxo3* in germ cells are distinctly highly expressed than those of granulosa cells. Thank you very much.

8. Fig. 7I: FOXO3 is expressed in the nuclei of inactive oocytes. It will only be translocated in the cytosol when oocytes are activated when primary follicles form. So most oocytes should have nuclear FOXO3. At P3, majority of the oocytes would have FOXO3 located in the nucleus and a few oocytes in the medulla should have FOXO3 located in the cytosol. Similarly, TAZ is a transcription factor. It is supposed be located in the nucleus.

**Reply:** Thank you, the comments are very great. We have reappraised the expressions of FOXO3 and TAZ in murine oocyte.

**1) FOXO3:** It has been established that FOXO3 plays a central role in maintenance of the dormant state, upon phosphorylation, FOXO3 is transported from the nucleus to the cytoplasm, thus triggering oocyte growth [1,2]. However, in perinatal mice at post-natal day (PD) 1, it is also reported that FOXO3 only located in the cytoplasm, at PD3, it mainly expressed in cytoplasm in the great majority of follicles, but partially translocated to the nucleus, and by PD14, it had completely translocated to the nucleus (see Ref 3 result Figs. 1A and 1B). Thus, we believe that FOXO3 protein located in the nucleus of majority oocytes would be acceptable.

**2) TAZ:** Accordingly, the location of TAZ protein in oocyte is inconclusive. With the immunohistochemical staining of Hippo signaling genes in ovaries of adult mice, their results showed TAZ were mainly in oocytes cytoplasm of primordial, primary, secondary, and antral follicles [4]. On the other hand, as transcriptional coactivators of TAZ, YAP1 was localized predominantly in the cytoplasm of oocytes from primordial to fully grown oocytes [5], similarly, YAP is barely detectable at E13.5, but is present in oocyte cytoplasm at E15.5, E18.5, and newborns ovaries [6], these results are anticipated in the cytoplasm of TAZ. However, in Sun’s report [5], they detected that “WWTR1 (also termed TAZ) was strongly nuclear in both the oocytes and granulosa cells from primordial to antral follicles, with the exception of oocytes in large follicles”. There are still doubts that TAZ is detected in the cytoplasm of oocyte at perinatal ovaries, and deeper study need to resolve these apparently conflicting results.

**Reference:**

[1] Shimamoto S, Nishimura Y, Nagamatsu G, et al. Hypoxia induces the dormant state in oocytes through expression of Foxo3 [J]. Proceedings of the National Academy of Sciences USA, 2019, 116(25): 12321-12326.

[2] Nagamatsu G, Shimamoto S, Hamazaki N, et al. Mechanical stress accompanied with nuclear rotation is involved in the dormant state of mouse oocytes [J]. Science Advances, 2019, 5(6): eaav9960.

[3] John G B, Gallardo T D, Shirley L J, et al. Foxo3 is a PI3K-dependent molecular switch controlling the initiation of oocyte growth [J]. Developmental Biology, 2008, 321(1): 197-204.

[4] Kawamura K, Cheng Y, Suzuki N, et al. Hippo signaling disruption and Akt stimulation of ovarian follicles for infertility treatment[J]. Proceedings of the National Academy of Sciences USA, 2013, 110(43): 17474-17479.

[5] Sun T, Pepling M E, Diaz F J. Lats1 deletion causes increased germ cell apoptosis and follicular cysts in mouse ovaries [J]. Biology of Reproduction, 2015, 93(1): 22, 1-11.

[6] Abbassi L, Malki S, Cockburn K, et al. Multiple mechanisms cooperate to constitutively exclude the transcriptional co-activator YAP from the nucleus during murine oogenesis[J]. Biology of Reproduction, 2016, 94(5): 102, 1-12.

9. Fig. S7 about analyses of other somatic cells is not mentioned at all in the results. This figure should be described in the result part. The rational of the authors to not focus on interstitium populations is: "However, due to the limited information available regarding other ovarian somatic compartments, the current study placed most attention on germ cells and granulosa cells." On the contrary, this actually would make a good argument to take advantage of these new single cell data to further study these cells populations that are less known than the germ cells and granulosa. Since the authors provide this figure S7, they should talk about it. This would bring more novelty to the study.

**Reply:** Your comments are very nice. As for the description of other somatic cell, the supplementary content has been added.

*“****Transcriptional signatures of ovarian somatic cells during primordial follicle assembly***

*Specially, the transcriptional signatures of ovarian somatic cell types were further explored. As shown in S7A Fig, the stromal cell line was clustered, and five clusters were produced, which all expressed the specific marker genes of stromal cells (S7B Fig), while the novel marker genes in each cell cluster were identified (S7C Fig). Additionally, the cell clustering analyses of endothelial cells (S7D Fig) and immune cells (S7E Fig) were performed, and three and four clusters were generated, respectively. More notably, compared to transcriptome patterns of stromal and endothelial cells, whose transcriptome indicated the distinct patterns dependent on pre- and post-natal manners, the transcriptional signature of immune cells was distinguished from them both, and it seemed to be more irregular. However, the potential roles of ovarian somatic cells participating in primordial follicle assembly still need to be addressed.”*

10. Most parts of the discussion are repetitions of results and including detailed lists of genes that should be only described in the result part. The discussion should be shorten and focus on the take-home messages and bigger picture of these results in the context of what is known in the field and what novelty this study provides.

**Reply:** Thanks, your comments are to the point. Here, we have made revision in the Discussion, the redundant content or repetitions of results have been properly handled, and the novel insights have been enhanced. Thanks again.
